# Classifying the Lifestyle of Metagenomically-Derived Phages Sequences Using Alignment-Free Methods

**DOI:** 10.3389/fmicb.2020.567769

**Published:** 2020-11-12

**Authors:** Kai Song

**Affiliations:** School of Mathematics and Statistics, Qingdao University, Qingdao, China

**Keywords:** alignment-free dissimilarity measures, Markov model, lytic phages, contigs, temperate phages

## Abstract

Phages are viruses that infect bacteria. The phages can be classified into two different categories based on their lifestyles: temperate and lytic. Now, the metavirome can generate a large number of fragments from the viral genomic sequences of entire environmental community, which makes it impossible to determine their lifestyles through experiments. Thus, there is a need to development computational methods for annotating phage contigs and making prediction of their lifestyles. Alignment-based methods for classifying phage lifestyle are limited by incomplete assembled genomes and nucleotide databases. Alignment-free methods based on the frequencies of *k*-mers were widely used for genome and metagenome comparison which did not rely on the completeness of genome or nucleotide databases. To mimic fragmented metagenomic sequences, the temperate and lytic phages genomic sequences were split into non-overlapping fragments with different lengths, then, I comprehensively compared nine alignment-free dissimilarity measures with a wide range of choices of *k*-mer length and Markov orders for predicting the lifestyles of these phage contigs. The dissimilarity measure, $d_{2}^{S}$, performed better than other dissimilarity measures for classifying the lifestyles of phages. Thus, I propose that the alignment-free method, $d_{2}^{S}$, can be used for predicting the lifestyles of phages which derived from the metagenomic data.

## Introduction

Viruses are distributed in every corner of the earth, and they play important roles in the ecosystem (Srinivasiah et al., 2008). A virus is a small individual with a simple structure, containing only one type of nucleic acid (DNA or RNA), and must parasitize and replicate in living cells (Whitman et al., 1998). Viruses can infect all kinds of organisms, from mammals to bacteria. An important class of viruses is bacteriophages, which can infect and kill bacterial cells.

The lifestyles of phages can be divided into two different types, temperate and lytic (Chopin et al., 2001; Knowles et al., 2016). Temperate phages can replicate and spread by integrating their genetic information into the bacterial genome. However, the lytic phages replicate themselves in bacterial cells and spread by killing the cells. Bacteriophages play important roles in microbial community, and identifying their lifestyles is the first step to understand their functions. Now, the metavirome can generate a large number of fragments from the viral genomic sequences of entire environmental community, which makes it impossible to determine their lifestyles through experiments. So, developing computational methods is necessary to predict the lifestyles of phages.

The previous studies of classifying phages using genomic data were mainly using alignment-based methods (Proux et al., 2002; Rohwer and Edwards, 2002; Lima-Mendez et al., 2008, 2011). However, very few studies focus on classifying the lifestyles of phages. McNair et al., 2012 utilizes a similarity algorithm and a supervised Random Forest classifier to predict the lifestyle of a phage (McNair et al., 2012). The similarity algorithm which creates a training set from phages with known lifestyles based on the alignment of protein sequences, is used to train a Random Forest to classify the lifestyle of a phage. Mavrich and Hatfull (2017) identified the temperate phages as those containing at least one temperate phage Pham (Mavrich and Hatfull, 2017). These two methods are based on protein sequence alignment, thus, the completely assembled phages sequences were needed before the usage of their methods. Nowadays, metavirome studies using high throughput sequencing technology can generate massive amounts of short read sequences from virus genomes (Lecuit and Eloit, 2013; Wylie et al., 2013; Brum et al., 2015). However, assembly of these short reads were difficulty for the highly mosaic organization of virus genomes (Hendrix et al., 1999). So, metavirome studies could produce large amount of incomplete of fragments from viral genome which made the previous alignment-based methods could not been used for predicting the lifestyles.

Alignment-free methods based on the frequencies of *k*-mers (*k*-words or *k*-tuples) were widely used for genome and metagenome comparison as recently reviewed (Song et al., 2014; Zielezinski et al., 2017; Ren et al., 2018). A *k*-tuple is a short base fragment of length *k* on genomic sequences. The alignment-free dissimilarity measures, $d_{2}^{S}$ and $d_{2}^{*}$, were firstly developed for comparing two long DNA sequences, and then, successfully applied in many other fields, including phylogenetic tree construction (Song et al., 2013), the comparison of metagenomic samples (Jiang et al., 2012; Liao et al., 2016; Song et al., 2019) and gene regulatory regions (Song et al., 2013), identification of horizontal gene transfer (Tang et al., 2018) and virus-host interactions (Ahlgren et al., 2017), and improving contig binning for metagenomes (Wang et al., 2017). Also, many other alignment-free methods have been developed and applicated in many fields, see the reviews (Zielezinski et al., 2017, 2019; Ren et al., 2018).

In this study, I have conducted a comprehensive evaluation of nine alignment-free dissimilarity measures over various *k*-mer lengths for classifying the lifestyles of phages. To evaluate prediction accuracy, I used a benchmark dataset of 1,562 phages genomes available at the National Center for Biotechnology Information (NCBI) for which the lifestyles were reported. Then, the 1,225 of the phages identified before 31/12/2013 were used for constructing the training models. The 337 of the phages identified between 1/1/2014 and 31/12/2016 were used for testing different alignment-free methods. Overall, the $d_{2}^{S}$ dissimilarity measure performed better than other dissimilarity measures for classifying the lifestyles of phages. The software is available at https://github.com/songkai1987/PhagePred.

## Materials and Methods

### Virus Databases

RefSeq genomes of phages infecting bacteria or archaea were downloaded from NCBI on 20/10/2019. The lifestyles for the 1,562 phages identified before 31/12/2016 were predicted in Mavrich and Hatfull (2017) (Mavrich and Hatfull, 2017). In the set of phages with a known lifestyle, there were 463 temperate phages and 1,099 lytic phages. The 1,225 phages identified before 31/12/2013 were used for constructing the training models (Supplementary Table 1). The 337 of the phages identified between 1/1/2014 and 31/12/2016 were used for testing different alignment-free dissimilarity measures (Supplementary Table 2). The 325 phages identified after 1/1/2017 were used for novel phages for testing (Supplementary Table 3). The lifestyles of these phages were predicted using the same methods in (Mavrich and Hatfull, 2017).

To mimic the phage contigs assembly from metagenomic data sets, the temperate and lytic phages genomic sequences were split into non-overlapping fragments with length *L* = 500, 1000, 3000, 5000, and 10,000 bp. Fragments were generated for phage genomes identified between 1 January 2014 and 31 December 2016 were used as testing sets (Table 1). To generate the evaluation datasets with the same proportion of temperate and lytic phage contigs, the same number of contigs were randomly sampled from the genomic sequences of lytic phage as the number of contigs from temperate phages.

**TABLE 1:** The number of fragments generated from the lytic and temperate phage genomes discovered between 1 January 2014 and 31 December 2016.

**Fragment length**	**Lytic**	**Temperate**
500 bp	68,815	13,657
1,000 bp	34,298	6,789
3,000 bp	11,278	2,217
5,000 bp	6,663	1,304
10,000 bp	3,217	621

### Alignment-Free Dissimilarity Measures

Several alignment-free dissimilarity measures based on genomic oligonucleotide frequencies have been developed to infer the relationship between genomic sequences. Here, I studied nine alignment-free measures based on two different principles—those that consider background frequencies of *k*-mers and those that do not. First, the *k*-mer frequencies from the phage genomic sequences identified before 31 December 2013 were extracted and merged as two training sets for temperate and lytic phages, respectively. Then, for a contig, its *k*-mer frequencies were also extracted and used for calculating its distance to temperate and lytic *k*-mer frequencies for inferring its lifestyle. Several common methods are used to calculate the distance: Euclidean distance (*Eu*), Manhattan distance (*Ma*), Chebyshev distance (*Ch*), and *d*_*2*_ (Blaisdell, 1986). The background normalization methods, including $d_{2}^{*}$, $d_{2}^{S}$ (Song et al., 2013), *CVTree* (Qi et al., 2004a,b), *Teeling* (Teeling et al., 2004), and *EuF* (Pride et al., 2006), which compute the expected *k*-mer frequencies to eliminate the effect of background and enhance the signal of differences between the viral sequences. These dissimilarity measures are described below.

Since a read could be from the forward or reverse strand of a genome, the read was considered together with its complement for calculating the occurrences of each *k*-mer. Thus, for a viral contig, all possible *k*-mers were calculated using a finite alphabet set *S* = {*A*,*C*,*G*,*T*}. For a given *k*-mer *w*, its occurrence in the contig is defined as *X*_*w*_ and the relative frequency of this *k*-mer is defined as $f_{w}^{X}=X_{w}\,/\,\sum_{w}X_{w}$. For a given *k*-mer *w* for temperate or lytic phages in training sets, its occurrence is defined as *Y*_*w*_.

Some dissimilarity measures, such as $d_{2}^{*}$ and $d_{2}^{S}$, need an *r*-th order Markov model for the background sequence. The expected number of occurrences of word *w* = *w*_1_*w*_2_⋯*w*_*k*_, *E*(*X*_*w*_), can be calculated using the Markov model. The transition probability matrix for the Markov model can be estimated based on the *r*-mers and (*r*-1)-mers, and the estimated probability of observing the *k*-mer *w*_1_*w*_2_⋯*w*_*r*_ is *P*_*M*_(*w*_*r* + 1_|*w*_1_*w*_2_⋯*w*_*r*_) = X_w_1 w_2 ⋯w_r+1_/X_w_1 w_2 ⋯w_r_. Then, *E*(*X*_*w*_) can be calculated as:

$$E\,\left(X_{w}\right)=\left(L-k+1\right)\,f_{w_{1}\,w_{2}\,\cdots \,w_{r}}^{X}\,\prod_{n=1}^{k-r}P_{M}\,\left(w_{n+r}|w_{n}\,w_{n+1}\,\cdots \,w_{n+r-1}\right)$$

where *L* is the length of the contig. The difference between the occurrences of *k*-mer *w* and its expected occurrences is defined ${\tilde{X}}_{w}=X_{w}-E\,\left(X_{w}\right)$.

The Euclidean distance is defined as:

$$E\,u=\sqrt{\sum_{w\in S^{k}}{\left|f_{w}^{X}-f_{w}^{Y}\right|}^{2}}$$

The Manhattan distance is defined as:

$$M\,a=\sum_{w\in S^{k}}\left|f_{w}^{X}-f_{w}^{Y}\right|$$

The Chebyshev distance is defined as:

$$C\,h=\max_{w\in S^{k}}\left|f_{w}^{X}-f_{w}^{X}\right|$$

The *d*_*2*_ dissimilarity measure is defined as:

$$d_{2}=\frac{1}{2}\,\left(1-\frac{\sum_{w\in S^{k}}X_{w}\,Y_{w}}{\sqrt{\sum_{w\in S^{k}}X_{w}^{2}}\,\sqrt{\sum_{w\in S^{k}}Y_{w}^{2}}}\right)$$

The $d_{2}^{*}$ dissimilarity measure is defined as:

$$d_{2}^{*}=\frac{1}{2}\,\left(1-\frac{\sum_{w}\frac{{\tilde{X}}_{w}}{\sqrt{E\,\left(X_{w}\right)}}\,\frac{{\tilde{Y}}_{w}}{\sqrt{E(Y_{w}})}}{\sqrt{\sum_{w}\frac{{\tilde{X}}_{w}^{2}}{E\,\left(X_{w}\right)}}\,\sqrt{\sum_{w}\frac{{\tilde{Y}}_{w}^{2}}{E\,\left(Y_{w}\right)}}}\right)$$

The $d_{2}^{S}$ dissimilarity measure is defined as:

$$d_{2}^{S}=\frac{1}{2}\,\left(1-\frac{\sum_{w\in S^{k}}\frac{{\tilde{X}}_{w}\,{\tilde{Y}}_{w}}{\sqrt{{\tilde{X}}_{w}^{2}+{\tilde{Y}}_{w}^{2}}}}{\sqrt{\sum_{w\in S^{k}}\frac{{\tilde{X}}_{w}^{2}}{\sqrt{{\tilde{X}}_{w}^{2}+{\tilde{Y}}_{w}^{2}}}}\,\sqrt{\sum_{w\in S^{k}}\frac{{\tilde{Y}}_{w}^{2}}{\sqrt{{\tilde{X}}_{w}^{2}+{\tilde{Y}}_{w}^{2}}}}}\right)$$

The *CVTree* dissimilarity measure is defined as:

$$C\,V\,T\,r\,e\,e=\frac{1}{2}\,\left(1-\frac{\sum_{w\in S^{k}}{\tilde{X}}_{w}\,{\tilde{Y}}_{w}}{\sqrt{\sum_{w\in S^{k}}{\tilde{X}}_{w}^{2}}\,\sqrt{\sum_{w\in S^{k}}{\tilde{Y}}_{w}^{2}}}\right)$$

where ${\tilde{X}}_{w}=X_{w}\,/\,E\,\left(X_{w}\right)$, *E*(*X*_*w*_) is estimated using a (*k*-2)-th order Markov model.

The *Teeling* dissimilarity measure is defined based on the (*k*-2)-th order Markov model:

$$T\,e\,e\,l\,i\,n\,g=\sum_{w\in S^{k}}\frac{X_{w}-E\,\left(X_{w}\right)}{\sqrt{v\,a\,r\,\left(X_{w}\right)}}\,\frac{Y_{w}-E\,\left(Y_{w}\right)}{\sqrt{v\,a\,r\,\left(Y_{w}\right)}}$$

where *E*(*X*_*w*_) and *var*(*X*_*w*_) for *w* = *w*_1_*w*_2_⋯*w*_*k*_ can be calculated as:

$$E\,\left(X_{w}\right)=\frac{X\,\left(w_{1}\,w_{2}\,\cdots \,w_{k-1}\right)\,X\,\left(w_{2}\,w_{3\,\cdots }\,w_{k}\right)}{X\,\left(w_{2}\,\cdots \,w_{k-1}\right)}$$

$$v\,a\,r\,\left(X_{w}\right)$$

$$=E\,{\left(X_{w}\right)}^{*}\,\frac{\begin{array}{c} \left(X\,\left(w_{2}\,\cdots \,w_{k-1}\right)-X\,\left(w_{1}\,w_{2}\,\cdots \,w_{k-1}\right)\right)\\ \left(X\,\left(w_{2}\,\cdots \,w_{k-1}\right)-X\,\left(w_{2}\,w_{3\,\cdots }\,w_{k}\right)\right) \end{array}}{\begin{array}{c} X\,{\left(w_{2}\,\cdots \,w_{k-1}\right)}^{2} \end{array}}$$

The *EuF* dissimilarity measure is also defined based on the (*k*-2)-th order Markov model:

$$E\,u\,F=\frac{1}{4^{k}}\,\sum_{w\in S^{4}}\left|{\tilde{X}}_{w}-{\tilde{Y}}_{w}\right|$$

where ${\tilde{X}}_{w}=X_{w}\,/\,E\,\left(X_{w}\right)$, *E*(*X*_*w*_) is estimated based on the (*k*-2)-th order Markov model as above.

## Results

**FIGURE 1:**
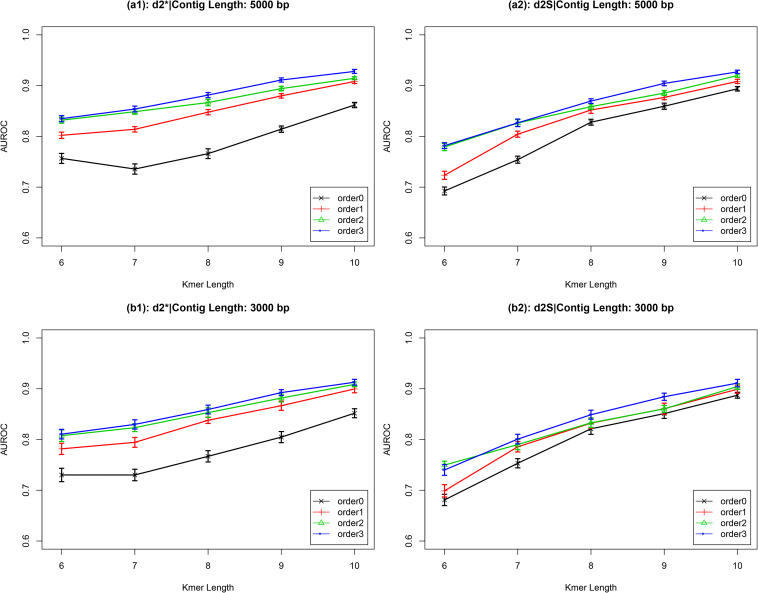
The work flow of my approach. First, the *k*-mer frequencies from two training sets of temperate and lytic phages were extracted, respectively. Then, the *k*-mer frequencies for a contig from new phage were extracted and used for calculating its distance to temperate and lytic *k*-mer frequencies for inferring its lifestyle.

The framework of my method is given in Figure 1. To test the performance of different alignment-free dissimilarity measures, two separate sets of temperate and lytic phage sequences were used for training and testing: temperate and lytic phage genomes sequenced before 31 December 2013 for training (Supplementary Table 1), after 1 January 2014 and before 31 December 2016 for testing (Supplementary Table 2). In order to evaluate the ability of these measures for classifying novel viruses based on the previous sequenced phage genomes, date was used for parting the training and testing sequences. To mimic fragmented metagenomic sequences, phage genomes in testing sets were split into non-overlapping fragments of various lengths *L* = 500, 1000, 3000, 5000, and 10,000 bp (Table 1).

### The Effects of *k*-mer Length, Markov Order, and Contig Length

**FIGURE 2:**
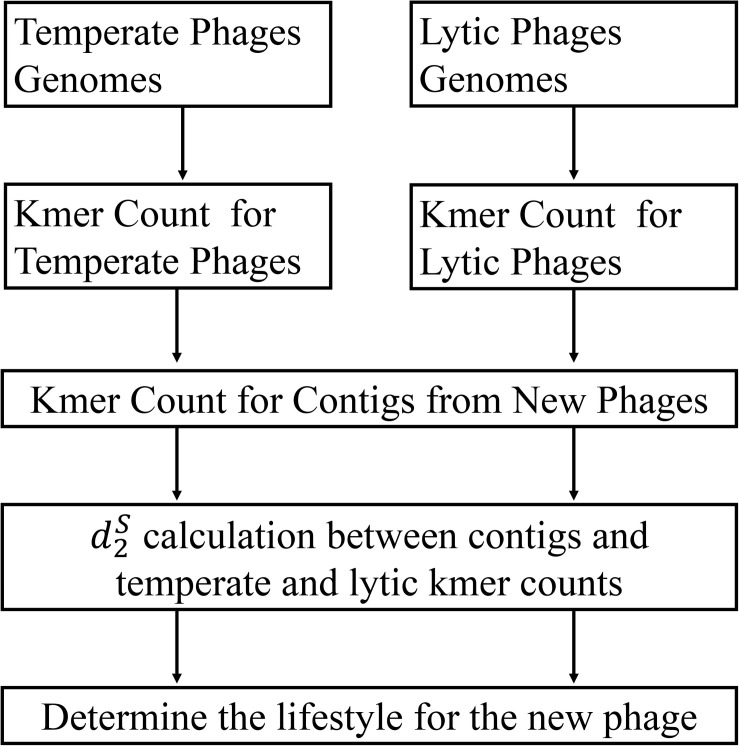
The AUROC values of $d_{2}^{*}$ and $d_{2}^{S}$ for classifying the lifestyles of phage contigs using *k*-mer lengths from 6 to 10, Markov order from 0 to 3, and contig lengths 5,000 bp (a) and 3,000 bp (b).

I used the temperate and lytic phages genomic sequences identified before 31 December 2013 to construct two *k*-mer frequency vectors, then calculated the distance (dissimilarity values) between a novel contig with these two *k*-mer frequency vectors. The ratio between the distance to temperate phages and to lytic phages which reflected the possibility of the contigs was temperate or not was calculated. The values lower than one indicated the contigs closer to the temperate phages and had higher possibility been from temperate phages. Then, the receiver operating characteristic (ROC) curves were used to evaluate $d_{2}^{*}$ and $d_{2}^{S}$’s performances for classification, while high values of the area under the ROC curves (AUROC) indicate good performance. For $d_{2}^{*}$ and $d_{2}^{S}$, AUROC values increased as *k*-mer length increased (Figure 2 and Supplementary Figure 1). For contigs with length ≥1,000 bp, AUROC values also increased as the Markov order of background sequences increased. These two dissimilarity measures, $d_{2}^{*}$ and $d_{2}^{S}$, had similar performance. For contigs with length ≥3,000 bp, the AUROC values were larger than 0.90 when the *k*-mer length was eight and Markov order was three. These high AUROC values demonstrate the strong ability of the $d_{2}^{*}$ and $d_{2}^{S}$ dissimilarity measures to correctly classifying newly obtained viral sequences. Based on these results, Markov order three was chosen for subsequent comparison with other alignment-free dissimilarity measures. To prove the validity of my proposed method, Supplementary Figure 2 showed that the distance of newly viral sequences to the temperate and lytic genomes.

### Comparison of These Alignment-Free Dissimilarity Measures’ Performance

I assessed the ability of the alignment-free dissimilarity measures, $d_{2}^{*}$ and $d_{2}^{S}$, to correctly classify phage contigs in comparison to other alignment-free dissimilarity measures. All these measures were tested using the same set of evaluation contigs as above: equal numbers of contigs subsampled from temperate and lytic phage genomes identified after 1 January 2014 and before 31 December 2016. AUROC values were scored for each of these measures using *k*-mer lengths from 6 to 10 and contig lengths 500 – 10,000 bp (Table 2 and Supplementary Table 4). AUROC values generally increased for all the measures when *k*-mer length was increased from 6 to 10. For contigs with length ≥1,000 bp, both of $d_{2}^{*}$ and $d_{2}^{S}$ had highest AUROC values, thus, outperform other dissimilarity measures. For contigs with length = 500 bp, all these measures had much lower AUROC values. The measures, Manhatten, Euclidean and EuF, had similar or a little better AUROC values as $d_{2}^{*}$ and $d_{2}^{S}$ when the *k*-mer length 10 for contig length 500 bp.

**TABLE 2:** The AUROC values of different dissimilarity measures for classifying the lifestyles of phage contigs using *k*-mer lengths from 6 to 10 and contig lengths 3,000 bp and 5,000 bp.

**Contig length 3000 bp**					
**K**	**6**	**7**	**8**	**9**	**10**
d2*	0.811	0.830	0.859	0.892	**0.913**
d2S	0.740	0.801	0.849	0.884	**0.911**
d2	0.766	0.784	0.804	0.830	0.855
Hao	0.773	0.721	0.759	0.739	0.735
Manhattan	0.698	0.718	0.794	0.836	0.869
Chebyshev	0.706	0.699	0.688	0.669	0.692
Euclidean	0.773	0.795	0.811	0.836	0.869
Teeling	0.779	0.727	0.756	0.738	0.739
EuF	0.762	0.728	0.810	0.858	0.896
**Contig length 5000 bp**					
K	6	7	8	9	10
d2*	0.835	0.854	0.881	0.911	**0.928**
d2S	0.782	0.827	0.870	0.904	**0.927**
d2	0.775	0.794	0.808	0.836	0.865
Hao	0.815	0.759	0.811	0.798	0.781
Manhattan	0.709	0.707	0.786	0.839	0.872
Chebyshev	0.723	0.723	0.710	0.688	0.687
Euclidean	0.779	0.799	0.820	0.841	0.876
Teeling	0.815	0.765	0.809	0.795	0.782
EuF	0.796	0.756	0.831	0.870	0.902

The background sequence Markov orders for $d_{2}^{*}$ and $d_{2}^{S}$ are fixed to three. The corresponding tables for cotig lengths 500, 1,000. and 10,000 bp are presented as Supplementary Table 4. The bold values represent the best results.

### Sensitivity of $d_{2}^{*}$ and $d_{2}^{S}$ to Mutations

Because of the alignment-free dissimilarity measures relies on nucleotide *k*-mer frequency and there are errors in sequencing technologies, the sensitivity of our newly developed alignment-free dissimilarity measures, $d_{2}^{*}$ and $d_{2}^{S}$, to mutations were tested. In Supplementary Figure 3, thirty replicates subsampled contigs with randomly inserted mutations at three different rates (0.001, 0.005, and 0.01) were used for comparing the performance with no mutations. The AUROC values were lower but not significantly for Markov order 0 and 1 at all the three different mutation rates. For Markov order 2 and 3, the AUROC values were only significantly lower at the highest rates of 0.01 mutations per bp (*P*-value < 0.01, *t*-test). As the sequencing error rates of Illumina and 454 platforms are ∼0.001 or 0.01, respectively (Glenn, 2011), sequencing errors only slightly impact the performance of the alignment-free dissimilarity measures for the NGS technologies.

### Assessment of the Classification of Novel Phages

To assess the ability of these alignment-free dissimilarity measures to classify novel phages, the 136 phages (Supplementary Table 5) (18 temperate phages and 108 lytic phages, identified after 1 January 2014 and before 31 December 2016) that had no significant nucleotide similarity (blastn search, *E*-values < 10−5) to previously phages genome sequences were used for testing. I classify these novel phages according to their distance to the temperate and lytic trained *k*-mer frequencies. The True Positive Rates (TPR) for temperate and lytic phages and the accuracy of classification for these phages were scored for these alignment-free dissimilarity measures using *k*-mer lengths from 6 to 10. I only showed the results that the TPRs for temperate and lytic phages were both larger than 60%. Table 3 showed that the best classification result was obtained by $d_{2}^{S}$ using *k*-mer length of 10 and Markov order of three. $d_{2}^{S}$ could correctly predicted 12 (66.7%) of temperate phages and 95 (87.9%) of lytic phages. For other alignment-free dissimilarity measures, the best classification result was obtained by Euclidean (Eu) distance using *k*-mer length of 10. Euclidean distance correctly predicted 11 (61.1%) of temperate phages and 91 (84.3%) of lytic phages.

**TABLE 3:** The True Positive Rates (TPR) for classifying the lifestyles for the 108 phages without significant nucleotide similarity to previously phages genome sequences using different dissimilarity measures.

	**K**	**Markov order**	**TPR1**	**TPR2**	**TPR**
d2S	6	0	0.611	0.750	0.730
d2S	6	3	0.778	0.611	0.635
d2S	7	0	0.667	0.769	0.754
d2S	7	1	0.667	0.676	0.675
d2S	7	3	0.611	0.611	0.611
d2S	8	1	0.667	0.722	0.714
d2S	8	2	0.889	0.806	0.817
d2S	8	3	0.722	0.722	0.722
d2S	9	3	0.667	0.778	0.762
d2S	10	3	0.667	0.879	0.849
d2	10		0.722	0.815	0.802
Chebyshev	7		0.889	0.630	0.667
Chebyshev	8		0.833	0.639	0.667
Euclidean	9		0.889	0.759	0.778
Euclidean	10		0.611	0.843	0.810

*The TPRs for temperate and lytic phages were both larger than 60% are shown in the Table. TPR1 is the Ture Positive Rate for temperate phages. TPR2 is the True Positive Rate for lytic phages. TPR is the Ture Positive Rate for all the phages.*

### Application to Classification of Phages Identified After January 2017

The 325 phage genomes identified after 1 January 2017 were downloaded for analysis (Supplementary Table 3). The lifestyle of these phages were predicted used the same method in Mavrich and Hatfull, 2017 (Mavrich and Hatfull, 2017), then 72 temperate phages and 253 lytic phages were identified. These phages were used for assessing the classification accuracy of the alignment-free dissimilarity measures. Table 4 showed that the best classification result was obtained by $d_{2}^{S}$ using *k*-mer length of 10 and Markov order of three. $d_{2}^{S}$ could correctly predicted 64 (88.9%) of temperate phages and 197 (77.9%) of lytic phages. For other alignment-free dissimilarity measures, the best classification results were obtained by *Teeling* and Euclidean (Eu) using *k*-mer length of 10. The dissimilarity measure of *Teeling* correctly predicted 63 (87.5%) of temperate phages and 187 (73.9%) of lytic phages. Euclidean distance correctly predicted 63 (87.5%) of temperate phages and 186 (73.5%) of lytic phages.

**TABLE 4:** The True Positive Rates (TPR) for classifying the lifestyles for the 325 phage genomes identified after 1 January 2017 using different dissimilarity measures.

	**K**	**Markov order**	**TPR1**	**TPR2**	**TPR**
d2S	6	2	0.833	0.704	0.732
d2S	7	2	0.986	0.601	0.686
d2S	7	3	0.903	0.625	0.686
d2S	8	2	0.958	0.660	0.726
d2S	8	3	0.917	0.700	0.748
d2S	9	1	0.736	0.763	0.757
d2S	9	2	0.889	0.676	0.723
d2S	9	3	0.639	0.806	0.769
d2S	10	1	0.736	0.810	0.794
d2S	10	2	0.778	0.787	0.785
d2S	10	3	0.889	0.779	0.803
d2	10		0.972	0.648	0.720
CVTree	7		0.931	0.680	0.735
Teeling	8		0.625	0.802	0.763
Teeling	9		0.875	0.739	0.769
Teeling	10		0.944	0.621	0.692
Euclidean	9		0.944	0.625	0.695
Euclidean	10		0.875	0.735	0.766

*The TPRs for temperate and lytic phages were both larger than 60% are shown in this Table. TPR1 is the Ture Positive Rate for temperate phages. TPR2 is the True Positive Rate for lytic phages. TPR is the Ture Positive Rate for all the phages.*

To mimic fragmented metagenomic sequences, these virus genomes were split into non-overlapping fragments of various length *L* = 1000, 3000, and 5000 bp. Table 5 showed that the best classification result was also obtained by $d_{2}^{S}$ using *k*-mer length of 10 and Markov order of three for contigs with length = 5000 bp. $d_{2}^{S}$ could correctly predicted 665 (86.4%) contigs from temperate phages and 3441 (81.6%) contigs from lytic phages. For contigs with length = 1000 and 3000 bp, $d_{2}^{S}$ also got the best classification results using *k*-mer length of 10 and Markov order of 3 (Supplementary Tables 6,7).

**TABLE 5:** The True Positive Rates (TPR) for classifying the lifestyles for contigs of 5,000 bp from the 325 phage genomes identified after 1 January 2017 using different dissimilarity measures.

	**K**	**Markov order**	**TPR1**	**TPR2**	**TPR**
d2S	6	2	0.701	0.678	0.681
d2S	7	2	0.771	0.681	0.695
d2S	8	2	0.840	0.719	0.738
d2S	8	3	0.718	0.780	0.770
d2S	9	1	0.909	0.689	0.723
d2S	9	2	0.926	0.753	0.779
d2S	9	3	0.638	0.826	0.797
d2S	10	1	0.773	0.826	0.817
d2S	10	2	0.864	0.806	0.815
d2S	10	3	0.851	0.816	0.821
d2	6		0.982	0.661	0.710
d2	7		0.978	0.674	0.721
d2	8		0.978	0.689	0.734
d2	9		0.969	0.717	0.756
d2	10		0.955	0.761	0.791
CVTree	7		0.783	0.708	0.720
Teeling	8		0.619	0.693	0.682
Teeling	9		0.666	0.638	0.642
Manhattan	6		0.975	0.646	0.696
Manhattan	7		0.970	0.698	0.740
Euclidean	6		0.973	0.664	0.712
Euclidean	7		0.962	0.680	0.724
Euclidean	8		0.955	0.707	0.745
Euclidean	9		0.930	0.747	0.775
Euclidean	10		0.848	0.799	0.807

*The TPRs for temperate and lytic phage contigs were both larger than 60% are shown in this Table. TPR1 is the Ture Positive Rate for temperate phage contigs. TPR2 is the True Positive Rate for lytic phage contigs. TPR is the Ture Positive Rate for all the phage contigs.*

## Discussion

In this study, I have conducted a comprehensive evaluation of nine alignment-free dissimilarity measures over various *k*-mer lengths for classifying the lifestyles of phages. For these dissimilarity measures requiring a background model, different orders of Markov chains were used for estimating background *k*-mer frequencies. These alignment-free dissimilarity measures, with a wide range of choices of *k*-mer length and Markov orders, were compared using the simulated metagenomic fragments of different length. The dissimilarity measure, $d_{2}^{S}$, could obtain the best performance for classifying the lifestyles of the phages contigs among these measures.

There are several limitations of the current study. First, for the dissimilarity measure, $d_{2}^{*}$, could obtain well performance as $d_{2}^{S}$ using the evaluation of ROC values, however, the performance for $d_{2}^{*}$ to classify novel phage contigs according to the distance to the temperate and lytic k-mer frequencies was very bad. The distribution of ratios between the distance to temperate phages and the distance to lytic phages calculated by $d_{2}^{*}$ was skewed to larger than one which reflect the systematic deviation in predicting the lifestyles for this dissimilarity measure. The unequal number of temperate and lytic phage genomes used in training set maybe cause this deviation for $d_{2}^{*}$. Second, the performance of these alignment-free dissimilarity measures depends on the phage genomes chosen in the training sets. In this study, I used the date as a criterion to split the phage genomes into training and testing sets. However, only less than two thousand phage genomes could be used in the study of these alignment-free measures which limits the accuracy of these methods. With the high-throughput sequencing technology widely used in viromics research, the assembled genomes for phages are becoming increasingly more available which would facilitate the development and application of these alignment-free dissimilarity measures. Third, the *k*-mer size *k* and orders of Markov models can markedly impact the performance of these alignment-free measures. In general, the *k*-mer size of 9 or 10 and Markov order of 2 or 3 for background sequences can give good performance. Since the viral genomes have great variability and highly mosaic organization, so longer length of *k*-mer and higher order of Markov chain can model the genomic sequences well. More studies are needed to see if this conclusion is robust for more phage genomes sequenced in the future.

In this study, I focused on classifying the lifestyles of phage contigs using alignment-free dissimilarity measures. Compared to alignment-based methods, the alignment-free methods can have better performance in classifying short contigs as a few kilobases without complete gene structure, however, alignment-free methods cannot give insights about the genome information responsible for the contigs. From this perspective, I can say that alignment-free and alignment-based methods for classifying phage contigs complement each other and should be used interactively for phage contigs classification.

## Data Availability Statement

The original contributions presented in the study are included in the article/Supplementary Material, further inquiries can be directed to the corresponding author.

## Author Contributions

KS conceived of the project, developed the methods, performed the computations, and contributed to the final manuscript.

## Conflict of Interest

The authors declare that the research was conducted in the absence of any commercial or financial relationships that could be construed as a potential conflict of interest.
