# Supplementary material for: Classifying the Lifestyle of Metagenomically-Derived Phages Sequences Using Alignment-Free Methods
Source: Front Microbiol. 2020 Nov 12;11:567769. doi: 10.3389/fmicb.2020.567769 (PMC7693541; doi:10.3389/fmicb.2020.567769)
Supplement: Supplementary file 1 [file Data_Sheet_1.docx]

Classifying the lifestyle of metagenomically-derived phages sequences using alignment-free methods Supplementary Material

Kai Song ^1,*^

1 School of Mathematics and Statistics, Qingdao University, Qingdao 266071, Shandong, China

*Author for Correspondence: Kai Song

Email: ksong@qdu.edu.cn

Figure S1. The AUROC values of $d_{2}^{*}$ and $d_{2}^{S}$ for classifying the lifestyles of phage contigs using *k*-mer lengths from 6 to 10, Markov order from 0 to 3, and contig lengths 10,000bp (a), 1,000 bp (b) and 500 bp (c).


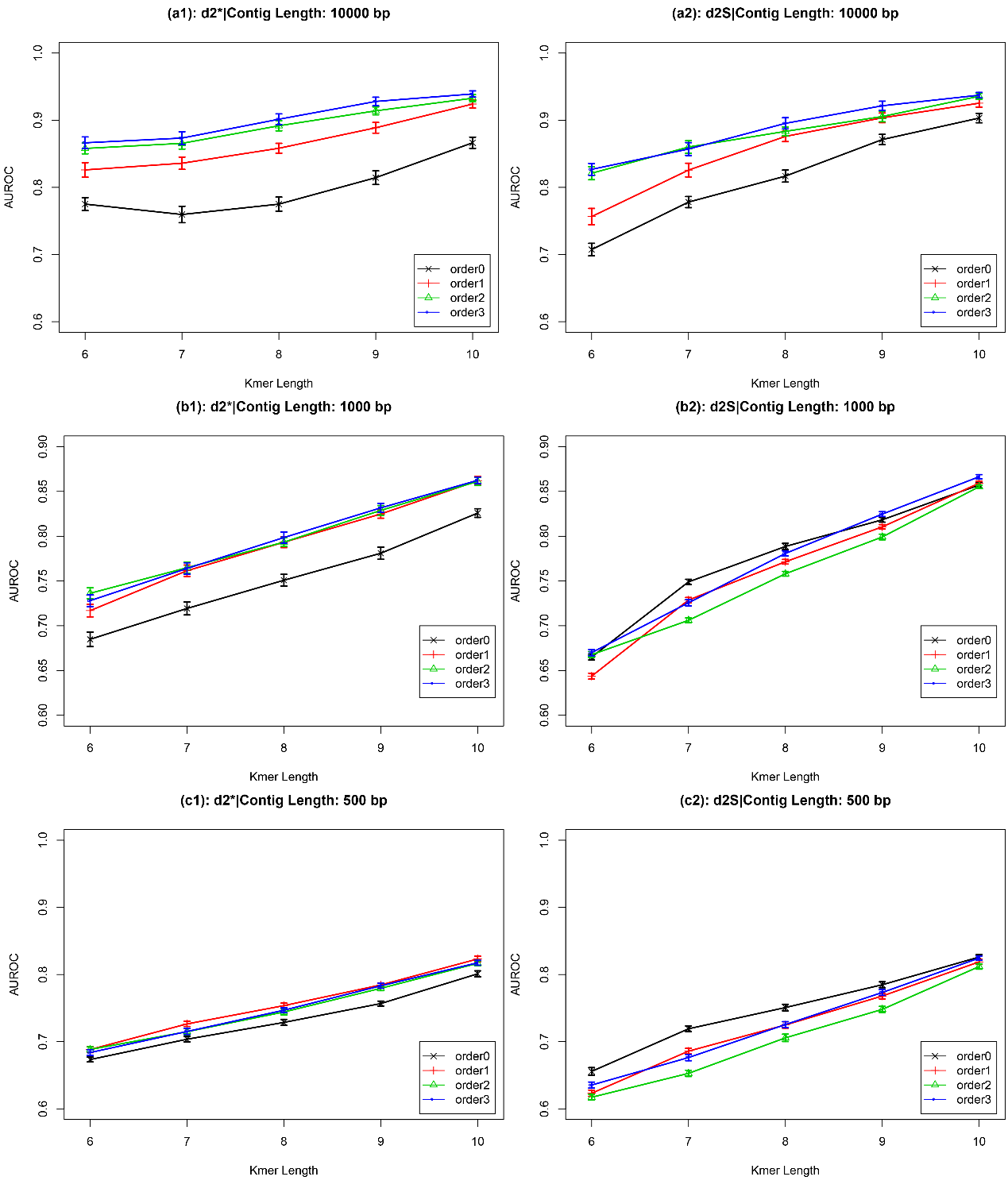


Figure S2. The distance of contigs from new phages to the temperate and lytic phages genomes. The black line represents the distance to temperate and lytic phages is equal.


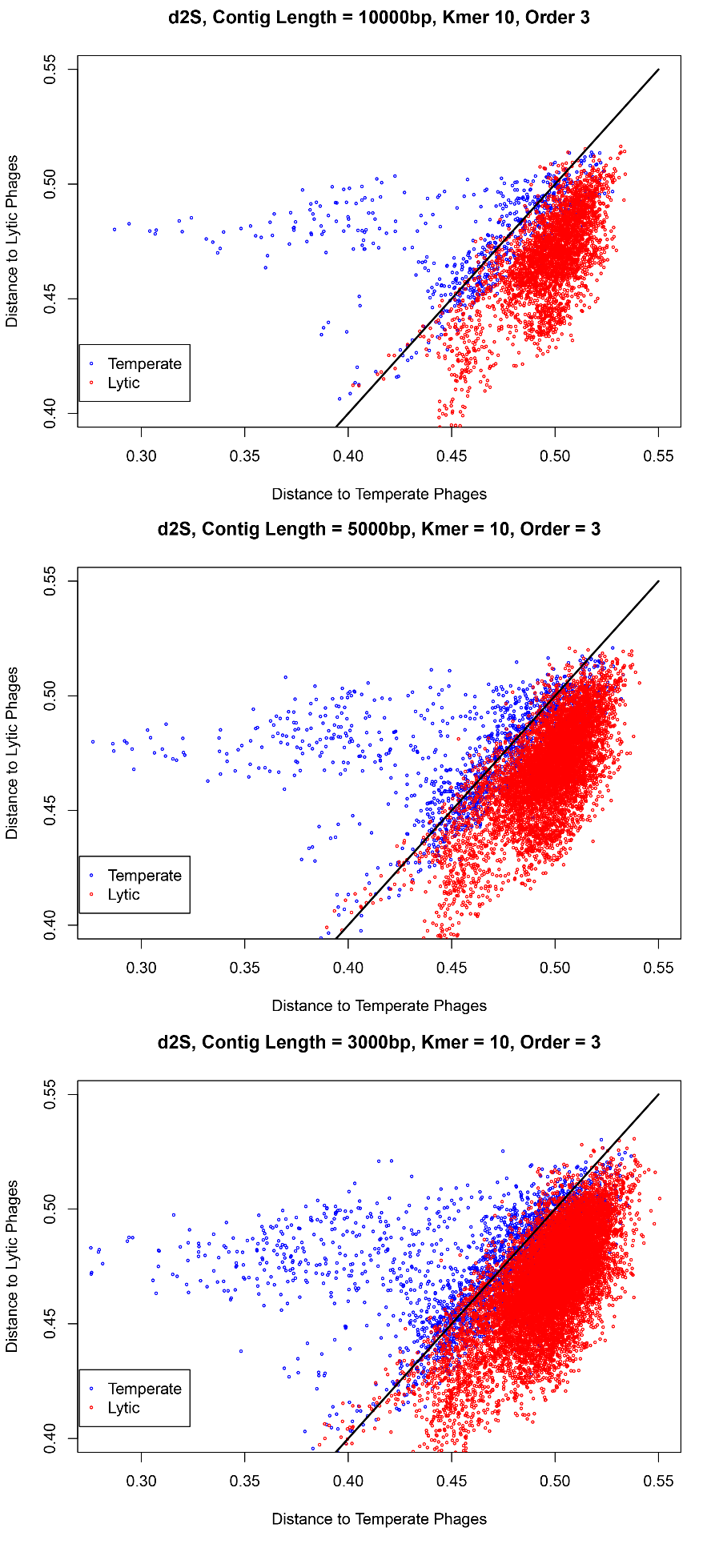


Figure S3. Sensitivity of $d_{2}^{*}$ and $d_{2}^{S}$ to random mutations applied to evaluation contigs. Prediction results as evaluated by AUROCs were determined on contigs from temperate and lytic phage genomes with no mutations applied vs. when random mutations were applied to the contigs at three different rates (0.001, 0.005, and 0.01 substitutions per position). Bars represent averages of 30 replicate datasets tested, and error bars indicate standard deviations.


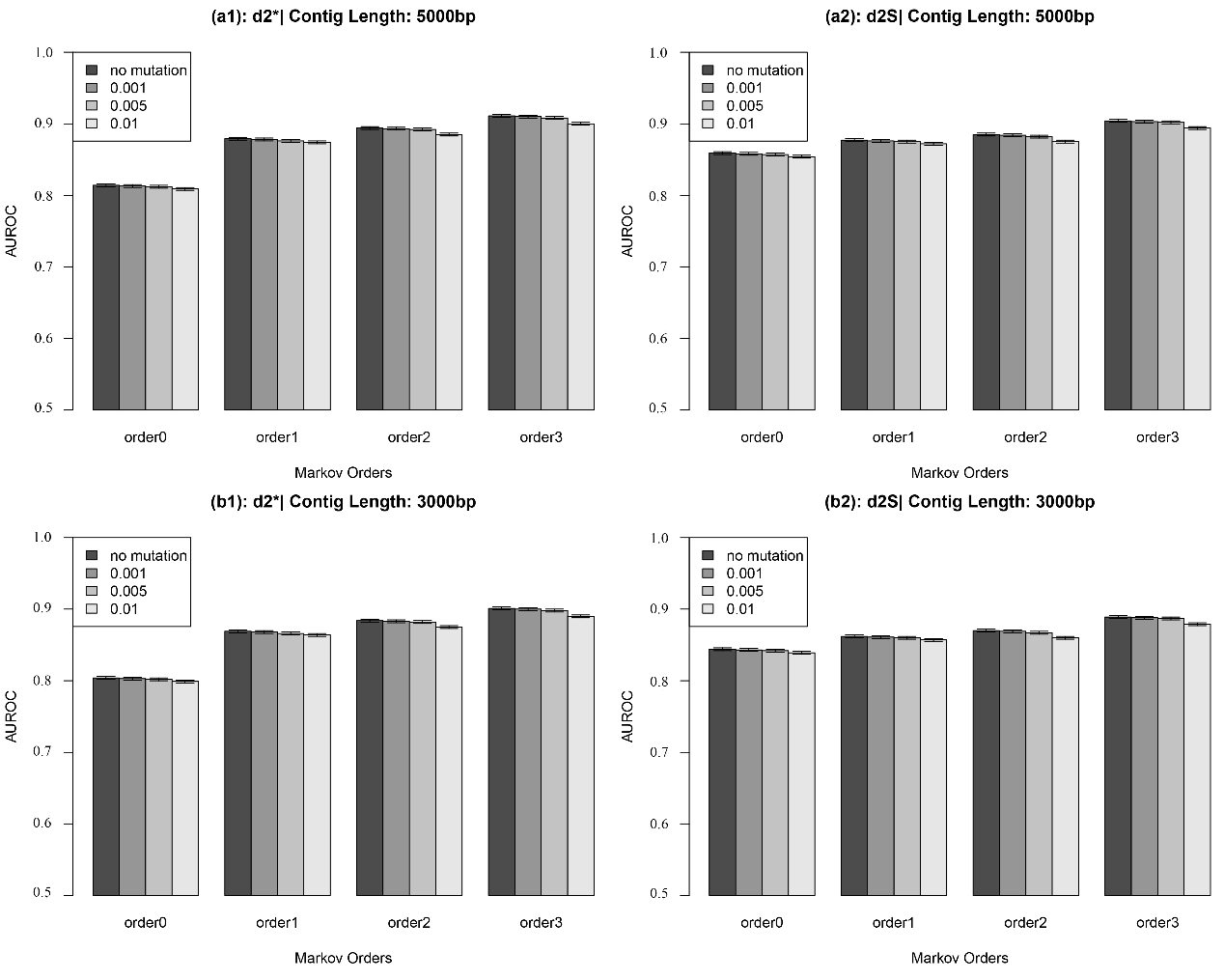


Table S1. The accession number of the 1,225 phages used for constructing the training models.

| Accession Number | Lifestyle | Accession Number | Lifestyle |
| --- | --- | --- | --- |
| NC_009760 | Lytic | NC_009737 | Temperate |
| NC_022343 | Lytic | NC_004615 | Temperate |
| NC_001741 | Lytic | NC_004617 | Temperate |
| NC_012558 | Lytic | NC_011103 | Temperate |
| NC_003793 | Lytic | NC_011357 | Temperate |
| NC_001365 | Lytic | NC_001317 | Temperate |
| NC_020843 | Lytic | NC_007047 | Temperate |
| NC_007807 | Lytic | NC_019512 | Temperate |
| NC_006356 | Lytic | NC_003291 | Temperate |
| NC_015250 | Lytic | NC_007051 | Temperate |
| NC_011045 | Lytic | NC_007061 | Temperate |
| NC_011703 | Lytic | NC_007055 | Temperate |
| NC_010576 | Lytic | NC_007052 | Temperate |
| NC_002194 | Lytic | NC_007054 | Temperate |
| NC_022323 | Lytic | NC_012753 | Temperate |
| NC_010821 | Lytic | NC_007062 | Temperate |
| NC_008208 | Lytic | NC_007049 | Temperate |
| NC_015249 | Lytic | NC_007060 | Temperate |
| NC_007019 | Lytic | NC_007048 | Temperate |
| NC_007022 | Lytic | NC_015938 | Temperate |
| NC_021853 | Lytic | NC_007059 | Temperate |
| NC_007053 | Lytic | NC_005356 | Temperate |
| NC_003438 | Lytic | NC_009526 | Temperate |
| NC_007461 | Lytic | NC_007050 | Temperate |
| NC_004678 | Lytic | NC_008464 | Temperate |
| NC_005135 | Lytic | NC_007063 | Temperate |
| NC_022968 | Lytic | NC_007064 | Temperate |
| NC_015251 | Lytic | NC_000924 | Temperate |
| NC_007046 | Lytic | NC_015263 | Temperate |
| NC_021867 | Lytic | NC_007057 | Temperate |
| NC_019519 | Lytic | NC_009815 | Temperate |
| NC_008370 | Lytic | NC_003216 | Temperate |
| NC_002185 | Lytic | NC_004112 | Temperate |
| NC_007806 | Lytic | NC_009810 | Temperate |
| NC_010353 | Lytic | NC_004827 | Temperate |
| NC_016657 | Lytic | NC_018283 | Temperate |
| NC_018088 | Lytic | NC_000935 | Temperate |
| NC_009811 | Lytic | NC_011551 | Temperate |
| NC_015253 | Lytic | NC_009812 | Temperate |
| NC_021337 | Lytic | NC_005887 | Temperate |
| NC_013645 | Lytic | NC_012743 | Temperate |
| NC_021316 | Lytic | NC_019917 | Temperate |
| NC_014660 | Lytic | NC_006557 | Temperate |
| NC_019444 | Lytic | NC_002666 | Temperate |
| NC_014661 | Lytic | NC_002667 | Temperate |
| NC_014663 | Lytic | NC_002668 | Temperate |
| NC_022328 | Lytic | NC_002669 | Temperate |
| NC_019725 | Lytic | NC_002670 | Temperate |
| NC_005260 | Lytic | NC_002671 | Temperate |
| NC_020879 | Lytic | NC_002796 | Temperate |
| NC_019543 | Lytic | NC_015254 | Temperate |
| NC_019923 | Lytic | NC_019912 | Temperate |
| NC_013693 | Lytic | NC_004813 | Temperate |
| NC_017969 | Lytic | NC_005357 | Temperate |
| NC_001330 | Lytic | NC_018085 | Temperate |
| NC_013598 | Lytic | NC_021533 | Temperate |
| NC_020478 | Lytic | NC_007581 | Temperate |
| NC_017984 | Lytic | NC_021324 | Temperate |
| NC_011523 | Lytic | NC_010355 | Temperate |
| NC_017688 | Lytic | NC_009514 | Temperate |
| NC_010363 | Lytic | NC_001396 | Temperate |
| NC_019916 | Lytic | NC_021338 | Temperate |
| NC_009643 | Lytic | NC_021318 | Temperate |
| NC_009813 | Lytic | NC_008722 | Temperate |
| NC_004165 | Lytic | NC_019507 | Temperate |
| NC_016770 | Lytic | NC_002484 | Temperate |
| NC_006548 | Lytic | NC_019544 | Temperate |
| NC_018863 | Lytic | NC_018452 | Temperate |
| NC_011222 | Lytic | NC_009552 | Temperate |
| NC_011040 | Lytic | NC_018859 | Temperate |
| NC_009990 | Lytic | NC_017732 | Temperate |
| NC_005258 | Lytic | NC_005294 | Temperate |
| NC_022331 | Lytic | NC_019934 | Temperate |
| NC_018856 | Lytic | NC_019927 | Temperate |
| NC_018086 | Lytic | NC_019932 | Temperate |
| NC_019515 | Lytic | NC_004775 | Temperate |
| NC_005263 | Lytic | NC_011976 | Temperate |
| NC_007497 | Lytic | NC_006949 | Temperate |
| NC_005262 | Lytic | NC_007056 | Temperate |
| NC_005342 | Lytic | NC_007805 | Temperate |
| NC_004333 | Lytic | NC_008193 | Temperate |
| NC_005886 | Lytic | NC_006552 | Temperate |
| NC_009015 | Lytic | NC_022750 | Temperate |
| NC_009447 | Lytic | NC_022753 | Temperate |
| NC_005882 | Lytic | NC_021774 | Temperate |
| NC_005091 | Lytic | NC_013059 | Temperate |
| NC_009604 | Lytic | NC_008376 | Temperate |
| NC_018860 | Lytic | NC_002166 | Temperate |
| NC_008694 | Lytic | NC_019768 | Temperate |
| NC_016764 | Lytic | NC_019710 | Temperate |
| NC_009799 | Lytic | NC_019717 | Temperate |
| NC_011046 | Lytic | NC_019714 | Temperate |
| NC_022769 | Lytic | NC_019769 | Temperate |
| NC_001909 | Lytic | NC_019767 | Temperate |
| NC_001629 | Lytic | NC_002730 | Temperate |
| NC_022773 | Lytic | NC_019711 | Temperate |
| NC_021861 | Lytic | NC_019723 | Temperate |
| NC_019500 | Lytic | NC_019719 | Temperate |
| NC_019541 | Lytic | NC_016158 | Temperate |
| NC_018857 | Lytic | NC_016160 | Temperate |
| NC_004814 | Lytic | NC_002167 | Temperate |
| NC_001706 | Lytic | NC_021864 | Temperate |
| NC_019449 | Lytic | NC_001697 | Temperate |
| NC_022761 | Lytic | NC_003315 | Temperate |
| NC_013155 | Lytic | NC_020482 | Temperate |
| NC_013152 | Lytic | NC_020483 | Temperate |
| NC_016570 | Lytic | NC_004914 | Temperate |
| NC_019538 | Lytic | NC_019501 | Temperate |
| NC_014662 | Lytic | NC_011612 | Temperate |
| NC_019406 | Lytic | NC_018281 | Temperate |
| NC_020844 | Lytic | NC_018284 | Temperate |
| NC_021537 | Lytic | NC_011614 | Temperate |
| NC_022974 | Lytic | NC_022756 | Temperate |
| NC_016161 | Lytic | NC_019456 | Temperate |
| NC_001825 | Lytic | NC_021773 | Temperate |
| NC_019457 | Lytic | NC_003313 | Temperate |
| NC_019933 | Lytic | NC_007924 | Temperate |
| NC_020205 | Lytic | NC_019928 | Temperate |
| NC_027997 | Lytic | NC_019931 | Temperate |
| NC_018861 | Lytic | NC_015266 | Temperate |
| NC_002180 | Lytic | NC_015273 | Temperate |
| NC_027996 | Lytic | NC_015265 | Temperate |
| NC_016562 | Lytic | NC_013055 | Temperate |
| NC_017974 | Lytic | NC_004745 | Temperate |
| NC_021531 | Lytic | NC_001447 | Temperate |
| NC_020479 | Lytic | NC_001416 | Temperate |
| NC_019540 | Lytic | NC_021556 | Temperate |
| NC_013594 | Lytic | NC_019486 | Temperate |
| NC_005178 | Lytic | NC_010179 | Temperate |
| NC_008717 | Lytic | NC_005354 | Temperate |
| NC_021068 | Lytic | NC_005355 | Temperate |
| NC_015274 | Lytic | NC_021539 | Temperate |
| NC_012697 | Lytic | NC_011104 | Temperate |
| NC_002072 | Lytic | NC_011801 | Temperate |
| NC_018854 | Lytic | NC_007809 | Temperate |
| NC_011042 | Lytic | NC_008562 | Temperate |
| NC_019485 | Lytic | NC_001942 | Temperate |
| NC_012696 | Lytic | NC_019706 | Temperate |
| NC_012419 | Lytic | NC_019720 | Temperate |
| NC_015270 | Lytic | NC_019715 | Temperate |
| NC_007623 | Lytic | NC_019708 | Temperate |
| NC_019524 | Lytic | NC_019704 | Temperate |
| NC_019539 | Lytic | NC_019721 | Temperate |
| NC_020477 | Lytic | NC_019716 | Temperate |
| NC_016566 | Lytic | NC_019709 | Temperate |
| NC_010583 | Lytic | NC_019705 | Temperate |
| NC_009014 | Lytic | NC_010237 | Temperate |
| NC_019509 | Lytic | NC_003050 | Temperate |
| NC_021563 | Lytic | NC_018282 | Temperate |
| NC_007810 | Lytic | NC_007967 | Temperate |
| NC_007814 | Lytic | NC_003085 | Temperate |
| NC_022744 | Lytic | NC_001901 | Temperate |
| NC_005282 | Lytic | NC_015252 | Temperate |
| NC_020480 | Lytic | NC_018835 | Temperate |
| NC_004306 | Lytic | NC_004303 | Temperate |
| NC_001956 | Lytic | NC_020874 | Temperate |
| NC_021775 | Lytic | NC_006882 | Temperate |
| NC_021772 | Lytic | NC_018846 | Temperate |
| NC_021782 | Lytic | NC_001895 | Temperate |
| NC_021779 | Lytic | NC_002371 | Temperate |
| NC_021780 | Lytic | NC_009803 | Temperate |
| NC_007066 | Lytic | NC_001609 | Temperate |
| NC_001420 | Lytic | NC_009804 | Temperate |
| NC_002649 | Lytic | NC_009819 | Temperate |
| NC_007458 | Lytic | NC_013195 | Temperate |
| NC_004665 | Lytic | NC_011373 | Temperate |
| NC_019448 | Lytic | NC_016564 | Temperate |
| NC_006945 | Lytic | NC_003324 | Temperate |
| NC_022766 | Lytic | NC_020858 | Temperate |
| NC_022790 | Lytic | NC_020856 | Temperate |
| NC_022771 | Lytic | NC_016767 | Temperate |
| NC_015293 | Lytic | NC_001331 | Temperate |
| NC_021067 | Lytic | NC_012756 | Temperate |
| NC_021073 | Lytic | NC_008723 | Temperate |
| NC_019724 | Lytic | NC_010945 | Temperate |
| NC_020484 | Lytic | NC_021784 | Temperate |
| NC_020481 | Lytic | NC_005284 | Temperate |
| NC_018855 | Lytic | NC_004167 | Temperate |
| NC_001332 | Lytic | NC_004616 | Temperate |
| NC_017983 | Lytic | NC_011344 | Temperate |
| NC_015157 | Lytic | NC_016762 | Temperate |
| NC_015158 | Lytic | NC_009018 | Temperate |
| NC_015159 | Lytic | NC_003524 | Temperate |
| NC_007856 | Lytic | NC_021800 | Temperate |
| NC_007817 | Lytic | NC_007145 | Temperate |
| NC_011167 | Lytic | NC_019921 | Temperate |
| NC_021783 | Lytic | NC_009235 | Temperate |
| NC_001954 | Lytic | NC_020199 | Temperate |
| NC_002014 | Lytic | NC_000896 | Temperate |
| NC_014260 | Lytic | NC_005893 | Temperate |
| NC_019503 | Lytic | NC_022791 | Temperate |
| NC_019423 | Lytic | NC_009231 | Temperate |
| NC_019416 | Lytic | NC_007917 | Temperate |
| NC_021540 | Lytic | NC_011398 | Temperate |
| NC_020203 | Lytic | NC_015568 | Temperate |
| NC_020198 | Lytic | NC_015262 | Temperate |
| NC_020202 | Lytic | NC_019506 | Temperate |
| NC_020200 | Lytic | NC_019496 | Temperate |
| NC_020204 | Lytic | NC_019508 | Temperate |
| NC_019726 | Lytic | NC_011318 | Temperate |
| NC_021777 | Lytic | NC_003278 | Temperate |
| NC_019450 | Lytic | NC_009236 | Temperate |
| NC_017674 | Lytic | NC_003309 | Temperate |
| NC_022916 | Lytic | NC_009234 | Temperate |
| NC_008371 | Lytic | NC_010324 | Temperate |
| NC_007291 | Lytic | NC_013696 | Temperate |
| NC_019419 | Lytic | NC_018454 | Temperate |
| NC_017975 | Lytic | NC_015295 | Temperate |
| NC_021860 | Lytic | NC_003288 | Temperate |
| NC_021854 | Lytic | NC_008798 | Temperate |
| NC_012741 | Lytic | NC_008799 | Temperate |
| NC_010105 | Lytic | NC_013646 | Temperate |
| NC_012740 | Lytic | NC_013643 | Temperate |
| NC_005880 | Lytic | NC_013648 | Temperate |
| NC_008152 | Lytic | NC_013644 | Temperate |
| NC_011043 | Lytic | NC_004305 | Temperate |
| NC_007637 | Lytic | NC_010342 | Temperate |
| NC_007456 | Lytic | NC_016568 | Temperate |
| NC_015719 | Lytic | NC_021865 | Temperate |
| NC_019410 | Lytic | NC_006938 | Temperate |
| NC_020865 | Lytic | NC_005857 | Temperate |
| NC_020854 | Lytic | NC_005822 | Temperate |
| NC_019420 | Lytic | NC_008201 | Temperate |
| NC_018278 | Lytic | NC_019421 | Temperate |
| NC_014036 | Lytic | NC_019422 | Temperate |
| NC_020080 | Lytic | NC_010147 | Temperate |
| NC_013647 | Lytic | NC_010808 | Temperate |
| NC_013649 | Lytic | NC_003157 | Temperate |
| NC_015272 | Lytic | NC_019418 | Temperate |
| NC_019935 | Lytic | NC_008583 | Temperate |
| NC_011216 | Lytic | NC_008617 | Temperate |
| NC_006294 | Lytic | NC_009542 | Temperate |
| NC_009817 | Lytic | NC_003356 | Temperate |
| NC_011534 | Lytic | NC_002486 | Temperate |
| NC_005083 | Lytic | NC_012784 | Temperate |
| NC_021317 | Lytic | NC_022914 | Temperate |
| NC_012530 | Lytic | NC_019502 | Temperate |
| NC_011165 | Lytic | NC_017978 | Temperate |
| NC_007501 | Lytic | NC_007902 | Temperate |
| NC_019454 | Lytic | NC_007804 | Temperate |
| NC_019925 | Lytic | NC_022757 | Temperate |
| NC_015585 | Lytic | NC_016765 | Temperate |
| NC_013692 | Lytic | NC_020841 | Temperate |
| NC_009936 | Lytic | NC_023006 | Temperate |
| NC_009935 | Lytic | NC_005340 | Temperate |
| NC_009554 | Lytic | NC_013021 | Temperate |
| NC_022989 | Lytic | NC_007045 | Temperate |
| NC_011166 | Lytic | NC_002321 | Temperate |
| NC_018273 | Lytic | NC_008689 | Temperate |
| NC_021787 | Lytic | NC_005069 | Temperate |
| NC_021785 | Lytic | NC_022330 | Temperate |
| NC_021781 | Lytic | NC_004302 | Temperate |
| NC_006565 | Lytic | NC_020839 | Temperate |
| NC_027298 | Lytic | NC_020489 | Temperate |
| NC_017972 | Lytic | NC_015466 | Temperate |
| NC_010326 | Lytic | NC_019488 | Temperate |
| NC_010325 | Lytic | NC_007058 | Temperate |
| NC_013691 | Lytic | NC_020866 | Temperate |
| NC_021344 | Lytic | NC_009382 | Temperate |
| NC_012884 | Lytic | NC_011589 | Temperate |
| NC_003287 | Lytic | NC_021801 | Temperate |
| NC_019407 | Lytic | NC_021863 | Temperate |
| NC_022772 | Lytic | NC_014460 | Temperate |
| NC_021070 | Lytic | NC_011802 | Temperate |
| NC_020857 | Lytic | NC_005344 | Temperate |
| NC_020847 | Lytic | NC_000872 | Temperate |
| NC_020845 | Lytic | NC_021857 | Temperate |
| NC_019443 | Lytic | NC_022749 | Temperate |
| NC_021336 | Lytic | NC_003444 | Temperate |
| NC_011085 | Lytic | NC_019489 | Temperate |
| NC_009818 | Lytic | NC_002661 | Temperate |
| NC_011613 | Lytic | NC_004996 | Temperate |
| NC_011611 | Lytic | NC_008721 | Temperate |
| NC_018274 | Lytic | NC_019513 | Temperate |
| NC_022746 | Lytic | NC_018277 | Temperate |
| NC_022091 | Lytic | NC_001884 | Temperate |
| NC_020082 | Lytic | NC_016761 | Temperate |
| NC_000929 | Lytic | NC_019545 | Temperate |
| NC_001341 | Lytic | NC_017985 | Temperate |
| NC_018837 | Lytic | NC_020872 | Temperate |
| NC_008720 | Lytic | NC_005841 | Temperate |
| NC_013651 | Lytic | NC_014900 | Temperate |
| NC_016658 | Lytic | NC_004313 | Temperate |
| NC_016659 | Lytic | NC_004348 | Temperate |
| NC_016569 | Lytic | NC_022767 | Temperate |
| NC_015464 | Lytic | NC_021326 | Temperate |
| NC_019451 | Lytic | NC_021323 | Temperate |
| NC_021529 | Lytic | NC_021332 | Temperate |
| NC_016571 | Lytic | NC_020490 | Temperate |
| NC_007709 | Lytic | NC_019915 | Temperate |
| NC_007710 | Lytic | NC_019914 | Temperate |
| NC_020878 | Lytic | NC_004913 | Temperate |
| NC_015280 | Lytic | NC_017973 | Temperate |
| NC_015284 | Lytic | NC_009531 | Temperate |
| NC_021071 | Lytic | NC_017968 | Temperate |
| NC_015283 | Lytic | NC_018264 | Temperate |
| NC_020855 | Lytic | NC_019445 | Temperate |
| NC_006883 | Lytic | NC_022776 | Temperate |
| NC_021559 | Lytic | NC_020197 | Temperate |
| NC_006884 | Lytic | NC_011645 | Temperate |
| NC_015290 | Lytic | NC_009761 | Temperate |
| NC_020835 | Lytic | NC_009762 | Temperate |
| NC_016656 | Lytic | NC_009763 | Temperate |
| NC_008363 | Lytic | NC_002747 | Temperate |
| NC_012663 | Lytic | NC_002703 | Temperate |
| NC_002515 | Lytic | NC_004066 | Temperate |
| NC_005856 | Lytic | NC_023007 | Temperate |
| NC_018272 | Lytic | NC_021325 | Temperate |
| NC_018271 | Lytic | NC_019402 | Temperate |
| NC_018269 | Lytic | NC_018275 | Temperate |
| NC_018280 | Lytic | NC_018279 | Temperate |
| NC_005131 | Lytic | NC_019722 | Temperate |
| NC_018276 | Lytic | NC_004456 | Temperate |
| NC_004746 | Lytic | NC_005879 | Temperate |
| NC_009814 | Lytic | NC_005891 | Temperate |
| NC_011308 | Lytic | NC_027981 | Temperate |
| NC_003390 | Lytic | NC_009016 | Temperate |
| NC_004679 | Lytic | NC_022747 | Temperate |
| NC_021852 | Lytic | NC_000902 | Temperate |
| NC_018831 | Lytic | NC_005345 | Temperate |
| NC_020880 | Lytic | NC_021334 | Temperate |
| NC_007808 | Lytic | NC_005056 | Temperate |
| NC_022096 | Lytic | NC_007065 | Temperate |
| NC_015294 | Lytic | NC_013599 | Temperate |
| NC_022967 | Lytic | NC_007024 | Temperate |
| NC_022970 | Lytic | NC_022758 | Temperate |
| NC_022986 | Lytic | NC_018285 | Temperate |
| NC_022966 | Lytic | NC_010116 | Temperate |
| NC_019913 | Lytic | NC_011356 | Temperate |
| NC_005884 | Lytic | NC_019522 | Temperate |
| NC_004466 | Lytic |  |  |
| NC_019521 | Lytic |  |  |
| NC_022982 | Lytic |  |  |
| NC_011810 | Lytic |  |  |
| NC_017976 | Lytic |  |  |
| NC_020853 | Lytic |  |  |
| NC_015297 | Lytic |  |  |
| NC_021342 | Lytic |  |  |
| NC_009551 | Lytic |  |  |
| NC_008367 | Lytic |  |  |
| NC_001418 | Lytic |  |  |
| NC_021558 | Lytic |  |  |
| NC_020079 | Lytic |  |  |
| NC_019452 | Lytic |  |  |
| NC_013638 | Lytic |  |  |
| NC_015785 | Lytic |  |  |
| NC_021062 | Lytic |  |  |
| NC_009821 | Lytic |  |  |
| NC_021802 | Lytic |  |  |
| NC_021791 | Lytic |  |  |
| NC_021797 | Lytic |  |  |
| NC_021805 | Lytic |  |  |
| NC_021803 | Lytic |  |  |
| NC_021806 | Lytic |  |  |
| NC_015721 | Lytic |  |  |
| NC_019525 | Lytic |  |  |
| NC_015208 | Lytic |  |  |
| NC_021795 | Lytic |  |  |
| NC_021798 | Lytic |  |  |
| NC_021790 | Lytic |  |  |
| NC_021794 | Lytic |  |  |
| NC_021799 | Lytic |  |  |
| NC_021789 | Lytic |  |  |
| NC_019523 | Lytic |  |  |
| NC_011048 | Lytic |  |  |
| NC_021796 | Lytic |  |  |
| NC_021804 | Lytic |  |  |
| NC_021788 | Lytic |  |  |
| NC_021792 | Lytic |  |  |
| NC_021793 | Lytic |  |  |
| NC_021855 | Lytic |  |  |
| NC_019911 | Lytic |  |  |
| NC_019924 | Lytic |  |  |
| NC_023693 | Lytic |  |  |
| NC_004777 | Lytic |  |  |
| NC_020081 | Lytic |  |  |
| NC_019782 | Lytic |  |  |
| NC_014635 | Lytic |  |  |
| NC_014636 | Lytic |  |  |
| NC_019528 | Lytic |  |  |
| NC_020862 | Lytic |  |  |
| NC_019405 | Lytic |  |  |
| NC_017980 | Lytic |  |  |
| NC_018083 | Lytic |  |  |
| NC_014457 | Lytic |  |  |
| NC_027353 | Lytic |  |  |
| NC_009237 | Lytic |  |  |
| NC_019926 | Lytic |  |  |
| NC_015292 | Lytic |  |  |
| NC_011811 | Lytic |  |  |
| NC_019929 | Lytic |  |  |
| NC_010106 | Lytic |  |  |
| NC_009904 | Lytic |  |  |
| NC_006953 | Lytic |  |  |
| NC_022971 | Lytic |  |  |
| NC_015264 | Lytic |  |  |
| NC_006936 | Lytic |  |  |
| NC_001730 | Lytic |  |  |
| NC_012418 | Lytic |  |  |
| NC_005045 | Lytic |  |  |
| NC_019520 | Lytic |  |  |
| NC_004629 | Lytic |  |  |
| NC_012742 | Lytic |  |  |
| NC_020870 | Lytic |  |  |
| NC_020083 | Lytic |  |  |
| NC_005964 | Lytic |  |  |
| NC_002643 | Lytic |  |  |
| NC_021856 | Lytic |  |  |
| NC_011142 | Lytic |  |  |
| NC_016163 | Lytic |  |  |
| NC_019909 | Lytic |  |  |
| NC_019919 | Lytic |  |  |
| NC_010807 | Lytic |  |  |
| NC_019530 | Lytic |  |  |
| NC_015586 | Lytic |  |  |
| NC_020860 | Lytic |  |  |
| NC_008265 | Lytic |  |  |
| NC_021569 | Lytic |  |  |
| NC_007189 | Lytic |  |  |
| NC_020842 | Lytic |  |  |
| NC_020201 | Lytic |  |  |
| NC_013697 | Lytic |  |  |
| NC_001422 | Lytic |  |  |
| NC_001271 | Lytic |  |  |
| NC_008584 | Lytic |  |  |
| NC_018270 | Lytic |  |  |
| NC_018084 | Lytic |  |  |
| NC_020883 | Lytic |  |  |
| NC_000867 | Lytic |  |  |
| NC_022770 | Lytic |  |  |
| NC_022751 | Lytic |  |  |
| NC_019542 | Lytic |  |  |
| NC_023005 | Lytic |  |  |
| NC_019447 | Lytic |  |  |
| NC_022987 | Lytic |  |  |
| NC_001421 | Lytic |  |  |
| NC_021331 | Lytic |  |  |
| NC_011107 | Lytic |  |  |
| NC_019529 | Lytic |  |  |
| NC_016071 | Lytic |  |  |
| NC_020863 | Lytic |  |  |
| NC_020846 | Lytic |  |  |
| NC_021534 | Lytic |  |  |
| NC_021561 | Lytic |  |  |
| NC_020849 | Lytic |  |  |
| NC_008364 | Lytic |  |  |
| NC_019490 | Lytic |  |  |
| NC_012638 | Lytic |  |  |
| NC_014467 | Lytic |  |  |
| NC_008515 | Lytic |  |  |
| NC_007023 | Lytic |  |  |
| NC_005066 | Lytic |  |  |
| NC_012635 | Lytic |  |  |
| NC_004928 | Lytic |  |  |
| NC_016165 | Lytic |  |  |
| NC_018832 | Lytic |  |  |
| NC_022765 | Lytic |  |  |
| NC_021300 | Lytic |  |  |
| NC_004735 | Lytic |  |  |
| NC_019408 | Lytic |  |  |
| NC_021560 | Lytic |  |  |
| NC_021557 | Lytic |  |  |
| NC_011201 | Lytic |  |  |
| NC_022917 | Lytic |  |  |
| NC_022915 | Lytic |  |  |
| NC_010811 | Lytic |  |  |
| NC_008574 | Lytic |  |  |
| NC_011399 | Lytic |  |  |
| NC_019548 | Lytic |  |  |
| NC_008575 | Lytic |  |  |
| NC_021866 | Lytic |  |  |
| NC_021862 | Lytic |  |  |
| NC_007603 | Lytic |  |  |
| NC_011041 | Lytic |  |  |
| NC_020837 | Lytic |  |  |
| NC_021530 | Lytic |  |  |
| NC_016164 | Lytic |  |  |
| NC_015463 | Lytic |  |  |
| NC_015465 | Lytic |  |  |
| NC_016766 | Lytic |  |  |
| NC_015569 | Lytic |  |  |
| NC_021536 | Lytic |  |  |
| NC_006820 | Lytic |  |  |
| NC_020859 | Lytic |  |  |
| NC_020486 | Lytic |  |  |
| NC_020867 | Lytic |  |  |
| NC_020838 | Lytic |  |  |
| NC_013085 | Lytic |  |  |
| NC_015281 | Lytic |  |  |
| NC_020851 | Lytic |  |  |
| NC_015282 | Lytic |  |  |
| NC_015279 | Lytic |  |  |
| NC_020875 | Lytic |  |  |
| NC_015289 | Lytic |  |  |
| NC_015287 | Lytic |  |  |
| NC_019516 | Lytic |  |  |
| NC_020869 | Lytic |  |  |
| NC_020416 | Lytic |  |  |
| NC_016565 | Lytic |  |  |
| NC_022920 | Lytic |  |  |
| NC_022918 | Lytic |  |  |
| NC_019511 | Lytic |  |  |
| NC_009875 | Lytic |  |  |
| NC_023009 | Lytic |  |  |
| NC_019549 | Lytic |  |  |
| NC_019550 | Lytic |  |  |
| NC_016763 | Lytic |  |  |
| NC_022752 | Lytic |  |  |
| NC_009232 | Lytic |  |  |
| NC_022754 | Lytic |  |  |
| NC_002214 | Lytic |  |  |
| NC_000871 | Lytic |  |  |
| NC_016073 | Lytic |  |  |
| NC_015456 | Lytic |  |  |
| NC_015457 | Lytic |  |  |
| NC_016567 | Lytic |  |  |
| NC_002519 | Lytic |  |  |
| NC_001835 | Lytic |  |  |
| NC_019910 | Lytic |  |  |
| NC_009987 | Lytic |  |  |
| NC_011756 | Lytic |  |  |
| NC_013600 | Lytic |  |  |
| NC_019487 | Lytic |  |  |
| NC_014595 | Lytic |  |  |
| NC_004831 | Lytic |  |  |
| NC_015269 | Lytic |  |  |
| NC_019417 | Lytic |  |  |
| NC_011421 | Lytic |  |  |
| NC_022763 | Lytic |  |  |
| NC_004166 | Lytic |  |  |
| NC_021868 | Lytic |  |  |
| NC_006940 | Lytic |  |  |
| NC_012223 | Lytic |  |  |
| NC_018843 | Lytic |  |  |
| NC_012868 | Lytic |  |  |
| NC_021343 | Lytic |  |  |
| NC_019455 | Lytic |  |  |
| NC_001270 | Lytic |  |  |
| NC_019411 | Lytic |  |  |
| NC_015288 | Lytic |  |  |
| NC_015286 | Lytic |  |  |
| NC_021072 | Lytic |  |  |
| NC_015285 | Lytic |  |  |
| NC_008296 | Lytic |  |  |
| NC_005833 | Lytic |  |  |
| NC_003298 | Lytic |  |  |
| NC_000866 | Lytic |  |  |
| NC_005859 | Lytic |  |  |
| NC_001604 | Lytic |  |  |
| NC_019446 | Lytic |  |  |
| NC_017971 | Lytic |  |  |
| NC_019442 | Lytic |  |  |
| NC_009540 | Lytic |  |  |
| NC_015937 | Lytic |  |  |
| NC_022088 | Lytic |  |  |
| NC_007021 | Lytic |  |  |
| NC_020414 | Lytic |  |  |
| NC_018850 | Lytic |  |  |
| NC_001998 | Lytic |  |  |
| NC_021532 | Lytic |  |  |
| NC_019527 | Lytic |  |  |
| NC_020873 | Lytic |  |  |
| NC_019398 | Lytic |  |  |
| NC_019400 | Lytic |  |  |
| NC_019401 | Lytic |  |  |
| NC_020078 | Lytic |  |  |
| NC_019504 | Lytic |  |  |
| NC_019510 | Lytic |  |  |
| NC_019514 | Lytic |  |  |
| NC_019399 | Lytic |  |  |
| NC_019517 | Lytic |  |  |
| NC_014792 | Lytic |  |  |
| NC_019403 | Lytic |  |  |
| NC_015933 | Lytic |  |  |
| NC_019404 | Lytic |  |  |
| NC_019718 | Lytic |  |  |
| NC_019526 | Lytic |  |  |
| NC_020871 | Lytic |  |  |
| NC_021063 | Lytic |  |  |
| NC_017864 | Lytic |  |  |
| NC_017865 | Lytic |  |  |
| NC_019918 | Lytic |  |  |
| NC_019813 | Lytic |  |  |
| NC_022090 | Lytic |  |  |
| NC_020877 | Lytic |  |  |
| NC_019518 | Lytic |  |  |
| NC_019713 | Lytic |  |  |
| NC_017981 | Lytic |  |  |
| NC_020850 | Lytic |  |  |
| NC_020868 | Lytic |  |  |
| NC_020848 | Lytic |  |  |
| NC_016162 | Lytic |  |  |
| NC_012757 | Lytic |  |  |
| NC_005948 | Lytic |  |  |
| NC_021562 | Lytic |  |  |
| NC_002362 | Lytic |  |  |
| NC_002363 | Lytic |  |  |
| NC_004736 | Lytic |  |  |
| NC_010495 | Lytic |  |  |
| NC_015271 | Lytic |  |  |
| NC_015296 | Lytic |  |  |
| NC_007149 | Lytic |  |  |
| NC_012662 | Lytic |  |  |
| NC_021776 | Lytic |  |  |
| NC_003907 | Lytic |  |  |
| NC_003327 | Lytic |  |  |
| NC_020488 | Lytic |  |  |
| NC_016563 | Lytic |  |  |
| NC_007821 | Lytic |  |  |
| NC_007734 | Lytic |  |  |
| NC_022094 | Lytic |  |  |
| NC_019505 | Lytic |  |  |
| NC_012749 | Lytic |  |  |
| NC_009543 | Lytic |  |  |
| NC_004902 | Lytic |  |  |
| NC_011038 | Lytic |  |  |
| NC_018087 | Lytic |  |  |

Table S2. The accession number of 337 phages used for testing.

| Accession Number | Lifestyle | Accession Number | Lifestyle |
| --- | --- | --- | --- |
| NC_028651 | Lytic | NC_028903 | Temperate |
| NC_023586 | Lytic | NC_023503 | Temperate |
| NC_025436 | Lytic | NC_028775 | Temperate |
| NC_025470 | Lytic | NC_028917 | Temperate |
| NC_025458 | Lytic | NC_024355 | Temperate |
| NC_025463 | Lytic | NC_029120 | Temperate |
| NC_027987 | Lytic | NC_028834 | Temperate |
| NC_025447 | Lytic | NC_029029 | Temperate |
| NC_027120 | Lytic | NC_023591 | Temperate |
| NC_028786 | Lytic | NC_027334 | Temperate |
| NC_029072 | Lytic | NC_028859 | Temperate |
| NC_024217 | Lytic | NC_028915 | Temperate |
| NC_025439 | Lytic | NC_029069 | Temperate |
| NC_029050 | Lytic | NC_028748 | Temperate |
| NC_029024 | Lytic | NC_024144 | Temperate |
| NC_025466 | Lytic | NC_025422 | Temperate |
| NC_029071 | Lytic | NC_022980 | Temperate |
| NC_029045 | Lytic | NC_024361 | Temperate |
| NC_029042 | Lytic | NC_025428 | Temperate |
| NC_029080 | Lytic | NC_024391 | Temperate |
| NC_024146 | Lytic | NC_029073 | Temperate |
| NC_025443 | Lytic | NC_025453 | Temperate |
| NC_024358 | Lytic | NC_028851 | Temperate |
| NC_028697 | Lytic | NC_026611 | Temperate |
| NC_027130 | Lytic | NC_028758 | Temperate |
| NC_026928 | Lytic | NC_028830 | Temperate |
| NC_023587 | Lytic | NC_024370 | Temperate |
| NC_027132 | Lytic | NC_028783 | Temperate |
| NC_029091 | Lytic | NC_029025 | Temperate |
| NC_029030 | Lytic | NC_004462 | Temperate |
| NC_027983 | Lytic | NC_027393 | Temperate |
| NC_025830 | Lytic | NC_029104 | Temperate |
| NC_028887 | Lytic | NC_028768 | Temperate |
| NC_024137 | Lytic | NC_024357 | Temperate |
| NC_027355 | Lytic | NC_023593 | Temperate |
| NC_024792 | Lytic | NC_027990 | Temperate |
| NC_027633 | Lytic | NC_026609 | Temperate |
| NC_027634 | Lytic | NC_024384 | Temperate |
| NC_027635 | Lytic | NC_024387 | Temperate |
| NC_027636 | Lytic | NC_027299 | Temperate |
| NC_027637 | Lytic | NC_028969 | Temperate |
| NC_027638 | Lytic | NC_026014 | Temperate |
| NC_024142 | Lytic | NC_028449 | Temperate |
| NC_029121 | Lytic | NC_028980 | Temperate |
| NC_023501 | Lytic | NC_028770 | Temperate |
| NC_024793 | Lytic | NC_024206 | Temperate |
| NC_024784 | Lytic | NC_028660 | Temperate |
| NC_024789 | Lytic | NC_028886 | Temperate |
| NC_028652 | Lytic | NC_029008 | Temperate |
| NC_028776 | Lytic | NC_021190 | Temperate |
| NC_024216 | Lytic | NC_023559 | Temperate |
| NC_025442 | Lytic | NC_026016 | Temperate |
| NC_023863 | Lytic | NC_028905 | Temperate |
| NC_027387 | Lytic | NC_028958 | Temperate |
| NC_028888 | Lytic | NC_029048 | Temperate |
| NC_028835 | Lytic | NC_028951 | Temperate |
| NC_025423 | Lytic | NC_028764 | Temperate |
| NC_023576 | Lytic | NC_028838 | Temperate |
| NC_023548 | Lytic | NC_029001 | Temperate |
| NC_024354 | Lytic | NC_029116 | Temperate |
| NC_023717 | Lytic | NC_028996 | Temperate |
| NC_024711 | Lytic | NC_023599 | Temperate |
| NC_015209 | Lytic | NC_029022 | Temperate |
| NC_027988 | Lytic | NC_022775 | Temperate |
| NC_027119 | Lytic | NC_028669 | Temperate |
| NC_029070 | Lytic | NC_023574 | Temperate |
| NC_023558 | Lytic | NC_029046 | Temperate |
| NC_024367 | Lytic | NC_028883 | Temperate |
| NC_028788 | Lytic | NC_028959 | Temperate |
| NC_028919 | Lytic | NC_028913 | Temperate |
| NC_028745 | Lytic | NC_028864 | Temperate |
| NC_028836 | Lytic | NC_024365 | Temperate |
| NC_028885 | Lytic | NC_027982 | Temperate |
| NC_028971 | Lytic | NC_023571 | Temperate |
| NC_027356 | Lytic | NC_023560 | Temperate |
| NC_029102 | Lytic | NC_025460 | Temperate |
| NC_028795 | Lytic | NC_027118 | Temperate |
| NC_024125 | Lytic | NC_025434 | Temperate |
| NC_024210 | Lytic | NC_028896 | Temperate |
| NC_023557 | Lytic | NC_028943 | Temperate |
| NC_023579 | Lytic | NC_029066 | Temperate |
| NC_023743 | Lytic | NC_023734 | Temperate |
| NC_027369 | Lytic | NC_027397 | Temperate |
| NC_027330 | Lytic | NC_029084 | Temperate |
| NC_025441 | Lytic | NC_028954 | Temperate |
| NC_025449 | Lytic | NC_029107 | Temperate |
| NC_025446 | Lytic | NC_025115 | Temperate |
| NC_027335 | Lytic | NC_025456 | Temperate |
| NC_025465 | Lytic | NC_025455 | Temperate |
| NC_029009 | Lytic | NC_025461 | Temperate |
| NC_029026 | Lytic | NC_029031 | Temperate |
| NC_023555 | Lytic | NC_013597 | Temperate |
| NC_024783 | Lytic | NC_029010 | Temperate |
| NC_029067 | Lytic | NC_029078 | Temperate |
| NC_024207 | Lytic | NC_029003 | Temperate |
| NC_028944 | Lytic | NC_028696 | Temperate |
| NC_028698 | Lytic | NC_028699 | Temperate |
| NC_023567 | Lytic | NC_029015 | Temperate |
| NC_027125 | Lytic | NC_028701 | Temperate |
| NC_025824 | Lytic | NC_027398 | Temperate |
| NC_027639 | Lytic | NC_028952 | Temperate |
| NC_027640 | Lytic | NC_027339 | Temperate |
| NC_027641 | Lytic | NC_028854 | Temperate |
| NC_027642 | Lytic | NC_022774 | Temperate |
| NC_027643 | Lytic | NC_023588 | Temperate |
| NC_027644 | Lytic | NC_029119 | Temperate |
| NC_027645 | Lytic | NC_027396 | Temperate |
| NC_027646 | Lytic | NC_028685 | Temperate |
| NC_027647 | Lytic | NC_023499 | Temperate |
| NC_027648 | Lytic | NC_023500 | Temperate |
| NC_028848 | Lytic | NC_028700 | Temperate |
| NC_023719 | Lytic | NC_028930 | Temperate |
| NC_028993 | Lytic | NC_027984 | Temperate |
| NC_023612 | Lytic | NC_027395 | Temperate |
| NC_029118 | Lytic | NC_028671 | Temperate |
| NC_025425 | Lytic | NC_028871 | Temperate |
| NC_028994 | Lytic | NC_028929 | Temperate |
| NC_023550 | Lytic | NC_028898 | Temperate |
| NC_029094 | Lytic | NC_028956 | Temperate |
| NC_028819 | Lytic | NC_028853 | Temperate |
| NC_027384 | Lytic | NC_028743 | Temperate |
| NC_024213 | Lytic | NC_025431 | Temperate |
| NC_028746 | Lytic | NC_023575 | Temperate |
| NC_027329 | Lytic | NC_026594 | Temperate |
| NC_024205 | Lytic | NC_025427 | Temperate |
| NC_027349 | Lytic | NC_028862 | Temperate |
| NC_028872 | Lytic | NC_023582 | Temperate |
| NC_024791 | Lytic | NC_028767 | Temperate |
| NC_028911 | Lytic | NC_028656 | Temperate |
| NC_023590 | Lytic | NC_025424 | Temperate |
| NC_023595 | Lytic | NC_027341 | Temperate |
| NC_026013 | Lytic | NC_024369 | Temperate |
| NC_029013 | Lytic | NC_028657 | Temperate |
| NC_028702 | Lytic |  |  |
| NC_023551 | Lytic |  |  |
| NC_024356 | Lytic |  |  |
| NC_028826 | Lytic |  |  |
| NC_029000 | Lytic |  |  |
| NC_025445 | Lytic |  |  |
| NC_027352 | Lytic |  |  |
| NC_024330 | Lytic |  |  |
| NC_028686 | Lytic |  |  |
| NC_029021 | Lytic |  |  |
| NC_029028 | Lytic |  |  |
| NC_028997 | Lytic |  |  |
| NC_028805 | Lytic |  |  |
| NC_029023 | Lytic |  |  |
| NC_028982 | Lytic |  |  |
| NC_023556 | Lytic |  |  |
| NC_029075 | Lytic |  |  |
| NC_027994 | Lytic |  |  |
| NC_027993 | Lytic |  |  |
| NC_028800 | Lytic |  |  |
| NC_027399 | Lytic |  |  |
| NC_028817 | Lytic |  |  |
| NC_028760 | Lytic |  |  |
| NC_029099 | Lytic |  |  |
| NC_029017 | Lytic |  |  |
| NC_024123 | Lytic |  |  |
| NC_024329 | Lytic |  |  |
| NC_025420 | Lytic |  |  |
| NC_025415 | Lytic |  |  |
| NC_025421 | Lytic |  |  |
| NC_029058 | Lytic |  |  |
| NC_029068 | Lytic |  |  |
| NC_028841 | Lytic |  |  |
| NC_024364 | Lytic |  |  |
| NC_024360 | Lytic |  |  |
| NC_024787 | Lytic |  |  |
| NC_027377 | Lytic |  |  |
| NC_027358 | Lytic |  |  |
| NC_024375 | Lytic |  |  |
| NC_024359 | Lytic |  |  |
| NC_024383 | Lytic |  |  |
| NC_024392 | Lytic |  |  |
| NC_026017 | Lytic |  |  |
| NC_028984 | Lytic |  |  |
| NC_029002 | Lytic |  |  |
| NC_022768 | Lytic |  |  |
| NC_025416 | Lytic |  |  |
| NC_024211 | Lytic |  |  |
| NC_030476 | Lytic |  |  |
| NC_025414 | Lytic |  |  |
| NC_027331 | Lytic |  |  |
| NC_024782 | Lytic |  |  |
| NC_025418 | Lytic |  |  |
| NC_028663 | Lytic |  |  |
| NC_028955 | Lytic |  |  |
| NC_024215 | Lytic |  |  |
| NC_024203 | Lytic |  |  |
| NC_025426 | Lytic |  |  |
| NC_024208 | Lytic |  |  |
| NC_028924 | Lytic |  |  |
| NC_028763 | Lytic |  |  |
| NC_024214 | Lytic |  |  |
| NC_013197 | Lytic |  |  |
| NC_023614 | Lytic |  |  |
| NC_029061 | Lytic |  |  |
| NC_028822 | Lytic |  |  |
| NC_028863 | Lytic |  |  |
| NC_029047 | Lytic |  |  |
| NC_028694 | Lytic |  |  |
| NC_023700 | Lytic |  |  |
| NC_027345 | Lytic |  |  |
| NC_028967 | Lytic |  |  |
| NC_027371 | Lytic |  |  |
| NC_022764 | Lytic |  |  |
| NC_025459 | Lytic |  |  |
| NC_028926 | Lytic |  |  |
| NC_028931 | Lytic |  |  |
| NC_028879 | Lytic |  |  |
| NC_028809 | Lytic |  |  |
| NC_029083 | Lytic |  |  |
| NC_028782 | Lytic |  |  |
| NC_027364 | Lytic |  |  |
| NC_028672 | Lytic |  |  |
| NC_024379 | Lytic |  |  |
| NC_029081 | Lytic |  |  |
| NC_028683 | Lytic |  |  |
| NC_026665 | Lytic |  |  |
| NC_023570 | Lytic |  |  |
| NC_027292 | Lytic |  |  |
| NC_023561 | Lytic |  |  |
| NC_025450 | Lytic |  |  |
| NC_028799 | Lytic |  |  |
| NC_028895 | Lytic |  |  |
| NC_025822 | Lytic |  |  |
| NC_025471 | Lytic |  |  |
| NC_028675 | Lytic |  |  |
| NC_028995 | Lytic |  |  |
| NC_029033 | Lytic |  |  |
| NC_029106 | Lytic |  |  |
| NC_028880 | Lytic |  |  |
| NC_028933 | Lytic |  |  |
| NC_029006 | Lytic |  |  |
| NC_027340 | Lytic |  |  |
| NC_023610 | Lytic |  |  |
| NC_028772 | Lytic |  |  |
| NC_028695 | Lytic |  |  |
| NC_028962 | Lytic |  |  |
| NC_028765 | Lytic |  |  |
| NC_022762 | Lytic |  |  |
| NC_024390 | Lytic |  |  |
| NC_024385 | Lytic |  |  |
| NC_024386 | Lytic |  |  |
| NC_024388 | Lytic |  |  |
| NC_024380 | Lytic |  |  |
| NC_024389 | Lytic |  |  |
| NC_024378 | Lytic |  |  |
| NC_027204 | Lytic |  |  |
| NC_028676 | Lytic |  |  |
| NC_028945 | Lytic |  |  |
| NC_028999 | Lytic |  |  |
| NC_024362 | Lytic |  |  |
| NC_023601 | Lytic |  |  |
| NC_023718 | Lytic |  |  |
| NC_023573 | Lytic |  |  |
| NC_029005 | Lytic |  |  |
| NC_028808 | Lytic |  |  |
| NC_028935 | Lytic |  |  |
| NC_027336 | Lytic |  |  |
| NC_027357 | Lytic |  |  |
| NC_027373 | Lytic |  |  |
| NC_027391 | Lytic |  |  |
| NC_027400 | Lytic |  |  |
| NC_027333 | Lytic |  |  |
| NC_027359 | Lytic |  |  |
| NC_027361 | Lytic |  |  |
| NC_027385 | Lytic |  |  |
| NC_027401 | Lytic |  |  |
| NC_027362 | Lytic |  |  |
| NC_027367 | Lytic |  |  |
| NC_027389 | Lytic |  |  |
| NC_027294 | Lytic |  |  |
| NC_027386 | Lytic |  |  |
| NC_027346 | Lytic |  |  |
| NC_027370 | Lytic |  |  |
| NC_027295 | Lytic |  |  |
| NC_027354 | Lytic |  |  |
| NC_023592 | Lytic |  |  |
| NC_027363 | Lytic |  |  |
| NC_027379 | Lytic |  |  |
| NC_027390 | Lytic |  |  |
| NC_023865 | Lytic |  |  |
| NC_027342 | Lytic |  |  |
| NC_028940 | Lytic |  |  |
| NC_027381 | Lytic |  |  |
| NC_028812 | Lytic |  |  |
| NC_028661 | Lytic |  |  |
| NC_029100 | Lytic |  |  |
| NC_023581 | Lytic |  |  |
| NC_024121 | Lytic |  |  |
| NC_023589 | Lytic |  |  |
| NC_026010 | Lytic |  |  |
| NC_025829 | Lytic |  |  |
| NC_027404 | Lytic |  |  |
| NC_027368 | Lytic |  |  |
| NC_023605 | Lytic |  |  |
| NC_023688 | Lytic |  |  |
| NC_029057 | Lytic |  |  |
| NC_028847 | Lytic |  |  |
| NC_025448 | Lytic |  |  |
| NC_025419 | Lytic |  |  |
| NC_027979 | Lytic |  |  |
| NC_025452 | Lytic |  |  |
| NC_029097 | Lytic |  |  |
| NC_027348 | Lytic |  |  |
| NC_027296 | Lytic |  |  |
| NC_024788 | Lytic |  |  |
| NC_025454 | Lytic |  |  |
| NC_023736 | Lytic |  |  |
| NC_028899 | Lytic |  |  |
| NC_028988 | Lytic |  |  |
| NC_029007 | Lytic |  |  |
| NC_028950 | Lytic |  |  |
| NC_025464 | Lytic |  |  |
| NC_023584 | Lytic |  |  |
| NC_028773 | Lytic |  |  |
| NC_027991 | Lytic |  |  |
| NC_028807 | Lytic |  |  |
| NC_027382 | Lytic |  |  |
| NC_028983 | Lytic |  |  |
| NC_025437 | Lytic |  |  |
| NC_028754 | Lytic |  |  |
| NC_023569 | Lytic |  |  |
| NC_028900 | Lytic |  |  |
| NC_028831 | Lytic |  |  |
| NC_028927 | Lytic |  |  |
| NC_028901 | Lytic |  |  |
| NC_028780 | Lytic |  |  |
| NC_028840 | Lytic |  |  |
| NC_028448 | Lytic |  |  |
| NC_028248 | Lytic |  |  |
| NC_027402 | Lytic |  |  |
| NC_023594 | Lytic |  |  |
| NC_027351 | Lytic |  |  |
| NC_028821 | Lytic |  |  |
| NC_027350 | Lytic |  |  |
| NC_027344 | Lytic |  |  |
| NC_026607 | Lytic |  |  |
| NC_028666 | Lytic |  |  |
| NC_028749 | Lytic |  |  |
| NC_028774 | Lytic |  |  |
| NC_025417 | Lytic |  |  |
| NC_028865 | Lytic |  |  |
| NC_028966 | Lytic |  |  |
| NC_023583 | Lytic |  |  |
| NC_028890 | Lytic |  |  |
| NC_027360 | Lytic |  |  |
| NC_028829 | Lytic |  |  |
| NC_025462 | Lytic |  |  |
| NC_025457 | Lytic |  |  |
| NC_028987 | Lytic |  |  |
| NC_028684 | Lytic |  |  |
| NC_028679 | Lytic |  |  |
| NC_028825 | Lytic |  |  |
| NC_027995 | Lytic |  |  |
| NC_024134 | Lytic |  |  |
| NC_024124 | Lytic |  |  |
| NC_024794 | Lytic |  |  |
| NC_028894 | Lytic |  |  |
| NC_028925 | Lytic |  |  |
| NC_028957 | Lytic |  |  |
| NC_028881 | Lytic |  |  |
| NC_025430 | Lytic |  |  |
| NC_027337 | Lytic |  |  |
| NC_024786 | Lytic |  |  |
| NC_024790 | Lytic |  |  |
| NC_024139 | Lytic |  |  |
| NC_025467 | Lytic |  |  |
| NC_028693 | Lytic |  |  |
| NC_028990 | Lytic |  |  |
| NC_029016 | Lytic |  |  |
| NC_028688 | Lytic |  |  |
| NC_028664 | Lytic |  |  |
| NC_028659 | Lytic |  |  |
| NC_028977 | Lytic |  |  |
| NC_028670 | Lytic |  |  |
| NC_028816 | Lytic |  |  |
| NC_028870 | Lytic |  |  |
| NC_028766 | Lytic |  |  |
| NC_023859 | Lytic |  |  |
| NC_028939 | Lytic |  |  |
| NC_026600 | Lytic |  |  |
| NC_026587 | Lytic |  |  |
| NC_026586 | Lytic |  |  |
| NC_028882 | Lytic |  |  |
| NC_024140 | Lytic |  |  |
| NC_026599 | Lytic |  |  |
| NC_026602 | Lytic |  |  |
| NC_027375 | Lytic |  |  |
| NC_026601 | Lytic |  |  |
| NC_028667 | Lytic |  |  |
| NC_024381 | Lytic |  |  |
| NC_026608 | Lytic |  |  |
| NC_028762 | Lytic |  |  |
| NC_028916 | Lytic |  |  |
| NC_023566 | Lytic |  |  |
| NC_025429 | Lytic |  |  |
| NC_023502 | Lytic |  |  |
| NC_023856 | Lytic |  |  |
| NC_024122 | Lytic |  |  |
| NC_023608 | Lytic |  |  |
| NC_024204 | Lytic |  |  |
| NC_029019 | Lytic |  |  |
| NC_028820 | Lytic |  |  |
| NC_028655 | Lytic |  |  |
| NC_025451 | Lytic |  |  |
| NC_028850 | Lytic |  |  |
| NC_029065 | Lytic |  |  |
| NC_024212 | Lytic |  |  |
| NC_023568 | Lytic |  |  |
| NC_028789 | Lytic |  |  |
| NC_026610 | Lytic |  |  |
| NC_025440 | Lytic |  |  |
| NC_024149 | Lytic |  |  |
| NC_026582 | Lytic |  |  |
| NC_026612 | Lytic |  |  |
| NC_026613 | Lytic |  |  |
| NC_028837 | Lytic |  |  |
| NC_027383 | Lytic |  |  |
| NC_023715 | Lytic |  |  |
| NC_029101 | Lytic |  |  |
| NC_027388 | Lytic |  |  |
| NC_024785 | Lytic |  |  |
| NC_028855 | Lytic |  |  |
| NC_027332 | Lytic |  |  |
| NC_029012 | Lytic |  |  |
| NC_029014 | Lytic |  |  |

Table S3. The accession number of 325 phages used for novel phages for testing.

| Accession Number | Lifestyle | Accession Number | Lifestyle |
| --- | --- | --- | --- |
| NC_041997 | Lytic | NC_041866 | Temperate |
| NC_042028 | Lytic | NC_041876 | Temperate |
| NC_041998 | Lytic | NC_042002 | Temperate |
| NC_041857 | Lytic | NC_042139 | Temperate |
| NC_041905 | Lytic | NC_041846 | Temperate |
| NC_041966 | Lytic | NC_041850 | Temperate |
| NC_041924 | Lytic | NC_041853 | Temperate |
| NC_041967 | Lytic | NC_041855 | Temperate |
| NC_041884 | Lytic | NC_041859 | Temperate |
| NC_041915 | Lytic | NC_041882 | Temperate |
| NC_041914 | Lytic | NC_041883 | Temperate |
| NC_042003 | Lytic | NC_041888 | Temperate |
| NC_042004 | Lytic | NC_041969 | Temperate |
| NC_042005 | Lytic | NC_041970 | Temperate |
| NC_042124 | Lytic | NC_041971 | Temperate |
| NC_042006 | Lytic | NC_041982 | Temperate |
| NC_042007 | Lytic | NC_041983 | Temperate |
| NC_042019 | Lytic | NC_041984 | Temperate |
| NC_042037 | Lytic | NC_041985 | Temperate |
| NC_042013 | Lytic | NC_041986 | Temperate |
| NC_040350 | Lytic | NC_041987 | Temperate |
| NC_040342 | Lytic | NC_041988 | Temperate |
| NC_040328 | Lytic | NC_041989 | Temperate |
| NC_040374 | Lytic | NC_042031 | Temperate |
| NC_040373 | Lytic | NC_042036 | Temperate |
| NC_040349 | Lytic | NC_042055 | Temperate |
| NC_040375 | Lytic | NC_042308 | Temperate |
| NC_041912 | Lytic | NC_042310 | Temperate |
| NC_042020 | Lytic | NC_042311 | Temperate |
| NC_041949 | Lytic | NC_042312 | Temperate |
| NC_041999 | Lytic | NC_042313 | Temperate |
| NC_041938 | Lytic | NC_042316 | Temperate |
| NC_041930 | Lytic | NC_042317 | Temperate |
| NC_041951 | Lytic | NC_042319 | Temperate |
| NC_041948 | Lytic | NC_042320 | Temperate |
| NC_042000 | Lytic | NC_042321 | Temperate |
| NC_041939 | Lytic | NC_042322 | Temperate |
| NC_041940 | Lytic | NC_042326 | Temperate |
| NC_041952 | Lytic | NC_042327 | Temperate |
| NC_041941 | Lytic | NC_042328 | Temperate |
| NC_041875 | Lytic | NC_042331 | Temperate |
| NC_041931 | Lytic | NC_042332 | Temperate |
| NC_041942 | Lytic | NC_042333 | Temperate |
| NC_041943 | Lytic | NC_042336 | Temperate |
| NC_041947 | Lytic | NC_042337 | Temperate |
| NC_041932 | Lytic | NC_042338 | Temperate |
| NC_042001 | Lytic | NC_042339 | Temperate |
| NC_042014 | Lytic | NC_041844 | Temperate |
| NC_041944 | Lytic | NC_041847 | Temperate |
| NC_042053 | Lytic | NC_041852 | Temperate |
| NC_041945 | Lytic | NC_041867 | Temperate |
| NC_042015 | Lytic | NC_041890 | Temperate |
| NC_041933 | Lytic | NC_041891 | Temperate |
| NC_041961 | Lytic | NC_041892 | Temperate |
| NC_041946 | Lytic | NC_041893 | Temperate |
| NC_041879 | Lytic | NC_041894 | Temperate |
| NC_042140 | Lytic | NC_041895 | Temperate |
| NC_043027 | Lytic | NC_041958 | Temperate |
| NC_041858 | Lytic | NC_042027 | Temperate |
| NC_041976 | Lytic | NC_042030 | Temperate |
| NC_041896 | Lytic | NC_042051 | Temperate |
| NC_042067 | Lytic | NC_042315 | Temperate |
| NC_041977 | Lytic | NC_042323 | Temperate |
| NC_042085 | Lytic | NC_042324 | Temperate |
| NC_042086 | Lytic | NC_042325 | Temperate |
| NC_042087 | Lytic | NC_042329 | Temperate |
| NC_042354 | Lytic | NC_042341 | Temperate |
| NC_042355 | Lytic | NC_042342 | Temperate |
| NC_041921 | Lytic | NC_042349 | Temperate |
| NC_042029 | Lytic | NC_041849 | Temperate |
| NC_042122 | Lytic | NC_041860 | Temperate |
| NC_041980 | Lytic | NC_042052 | Temperate |
| NC_042057 | Lytic |  |  |
| NC_041959 | Lytic |  |  |
| NC_042125 | Lytic |  |  |
| NC_042101 | Lytic |  |  |
| NC_041960 | Lytic |  |  |
| NC_042021 | Lytic |  |  |
| NC_042022 | Lytic |  |  |
| NC_042023 | Lytic |  |  |
| NC_042127 | Lytic |  |  |
| NC_042126 | Lytic |  |  |
| NC_041978 | Lytic |  |  |
| NC_041972 | Lytic |  |  |
| NC_042098 | Lytic |  |  |
| NC_042056 | Lytic |  |  |
| NC_041973 | Lytic |  |  |
| NC_042018 | Lytic |  |  |
| NC_041974 | Lytic |  |  |
| NC_041975 | Lytic |  |  |
| NC_041979 | Lytic |  |  |
| NC_041863 | Lytic |  |  |
| NC_041864 | Lytic |  |  |
| NC_041869 | Lytic |  |  |
| NC_042046 | Lytic |  |  |
| NC_041919 | Lytic |  |  |
| NC_041906 | Lytic |  |  |
| NC_041936 | Lytic |  |  |
| NC_041920 | Lytic |  |  |
| NC_041874 | Lytic |  |  |
| NC_041873 | Lytic |  |  |
| NC_041898 | Lytic |  |  |
| NC_041897 | Lytic |  |  |
| NC_041871 | Lytic |  |  |
| NC_041935 | Lytic |  |  |
| NC_042043 | Lytic |  |  |
| NC_041990 | Lytic |  |  |
| NC_041937 | Lytic |  |  |
| NC_042084 | Lytic |  |  |
| NC_041926 | Lytic |  |  |
| NC_042307 | Lytic |  |  |
| NC_041845 | Lytic |  |  |
| NC_041872 | Lytic |  |  |
| NC_041918 | Lytic |  |  |
| NC_042083 | Lytic |  |  |
| NC_042108 | Lytic |  |  |
| NC_042088 | Lytic |  |  |
| NC_041950 | Lytic |  |  |
| NC_042045 | Lytic |  |  |
| NC_042089 | Lytic |  |  |
| NC_042132 | Lytic |  |  |
| NC_041886 | Lytic |  |  |
| NC_042102 | Lytic |  |  |
| NC_042123 | Lytic |  |  |
| NC_041981 | Lytic |  |  |
| NC_042093 | Lytic |  |  |
| NC_041900 | Lytic |  |  |
| NC_042041 | Lytic |  |  |
| NC_041899 | Lytic |  |  |
| NC_042024 | Lytic |  |  |
| NC_040341 | Lytic |  |  |
| NC_041862 | Lytic |  |  |
| NC_040329 | Lytic |  |  |
| NC_042099 | Lytic |  |  |
| NC_042109 | Lytic |  |  |
| NC_042117 | Lytic |  |  |
| NC_042110 | Lytic |  |  |
| NC_042111 | Lytic |  |  |
| NC_042118 | Lytic |  |  |
| NC_042119 | Lytic |  |  |
| NC_041965 | Lytic |  |  |
| NC_042034 | Lytic |  |  |
| NC_042050 | Lytic |  |  |
| NC_041901 | Lytic |  |  |
| NC_042032 | Lytic |  |  |
| NC_041993 | Lytic |  |  |
| NC_042033 | Lytic |  |  |
| NC_042035 | Lytic |  |  |
| NC_042330 | Lytic |  |  |
| NC_042309 | Lytic |  |  |
| NC_042340 | Lytic |  |  |
| NC_042318 | Lytic |  |  |
| NC_042334 | Lytic |  |  |
| NC_042314 | Lytic |  |  |
| NC_042335 | Lytic |  |  |
| NC_043767 | Lytic |  |  |
| NC_042058 | Lytic |  |  |
| NC_041909 | Lytic |  |  |
| NC_042120 | Lytic |  |  |
| NC_041868 | Lytic |  |  |
| NC_044940 | Lytic |  |  |
| NC_041878 | Lytic |  |  |
| NC_041854 | Lytic |  |  |
| NC_042131 | Lytic |  |  |
| NC_041954 | Lytic |  |  |
| NC_041955 | Lytic |  |  |
| NC_041956 | Lytic |  |  |
| NC_041957 | Lytic |  |  |
| NC_042090 | Lytic |  |  |
| NC_041925 | Lytic |  |  |
| NC_041913 | Lytic |  |  |
| NC_042121 | Lytic |  |  |
| NC_042103 | Lytic |  |  |
| NC_041934 | Lytic |  |  |
| NC_042107 | Lytic |  |  |
| NC_041994 | Lytic |  |  |
| NC_041903 | Lytic |  |  |
| NC_041907 | Lytic |  |  |
| NC_041902 | Lytic |  |  |
| NC_041953 | Lytic |  |  |
| NC_042343 | Lytic |  |  |
| NC_042104 | Lytic |  |  |
| NC_042081 | Lytic |  |  |
| NC_041877 | Lytic |  |  |
| NC_041885 | Lytic |  |  |
| NC_041904 | Lytic |  |  |
| NC_042091 | Lytic |  |  |
| NC_041881 | Lytic |  |  |
| NC_041865 | Lytic |  |  |
| NC_041880 | Lytic |  |  |
| NC_041964 | Lytic |  |  |
| NC_042092 | Lytic |  |  |
| NC_041870 | Lytic |  |  |
| NC_042080 | Lytic |  |  |
| NC_042079 | Lytic |  |  |
| NC_041968 | Lytic |  |  |
| NC_042054 | Lytic |  |  |
| NC_041851 | Lytic |  |  |
| NC_041911 | Lytic |  |  |
| NC_041908 | Lytic |  |  |
| NC_034248 | Lytic |  |  |
| NC_042049 | Lytic |  |  |
| NC_041963 | Lytic |  |  |
| NC_042040 | Lytic |  |  |
| NC_041887 | Lytic |  |  |
| NC_042350 | Lytic |  |  |
| NC_042096 | Lytic |  |  |
| NC_042097 | Lytic |  |  |
| NC_042044 | Lytic |  |  |
| NC_041923 | Lytic |  |  |
| NC_042025 | Lytic |  |  |
| NC_041922 | Lytic |  |  |
| NC_041991 | Lytic |  |  |
| NC_041992 | Lytic |  |  |
| NC_042346 | Lytic |  |  |
| NC_041917 | Lytic |  |  |
| NC_041996 | Lytic |  |  |
| NC_042047 | Lytic |  |  |
| NC_042017 | Lytic |  |  |
| NC_042075 | Lytic |  |  |
| NC_042076 | Lytic |  |  |
| NC_042077 | Lytic |  |  |
| NC_042039 | Lytic |  |  |
| NC_042078 | Lytic |  |  |
| NC_041995 | Lytic |  |  |
| NC_041929 | Lytic |  |  |
| NC_041927 | Lytic |  |  |
| NC_041928 | Lytic |  |  |
| NC_042082 | Lytic |  |  |
| NC_042105 | Lytic |  |  |
| NC_042106 | Lytic |  |  |
| NC_042016 | Lytic |  |  |
| NC_042008 | Lytic |  |  |
| NC_042009 | Lytic |  |  |
| NC_042010 | Lytic |  |  |
| NC_042011 | Lytic |  |  |
| NC_041889 | Lytic |  |  |
| NC_042012 | Lytic |  |  |
| NC_042042 | Lytic |  |  |
| NC_041856 | Lytic |  |  |
| NC_041962 | Lytic |  |  |
| NC_042100 | Lytic |  |  |
| NC_042094 | Lytic |  |  |
| NC_042074 | Lytic |  |  |
| NC_041910 | Lytic |  |  |
| NC_042095 | Lytic |  |  |
| NC_041916 | Lytic |  |  |
| NC_043028 | Lytic |  |  |
| NC_030928 | Lytic |  |  |
| NC_030937 | Lytic |  |  |
| NC_042345 | Lytic |  |  |
| NC_042344 | Lytic |  |  |
| NC_042116 | Lytic |  |  |

Table S4. The AUROC values of different dissimilarity measures for classifying the lifestyles of phage contigs using *k*-mer lengths from 6 to 10 and contig lengths 500bp, 1,000bp and 5,000 bp. The background sequence Markov orders for $d_{2}^{*}$ and $d_{2}^{S}$ are fixed to three.

| Contig Length 500 bp | | | | | |
| --- | --- | --- | --- | --- | --- |
| K | 6 | 7 | 8 | 9 | 10 |
| d2* | 0.684 | 0.715 | 0.747 | 0.783 | 0.817 |
| d2S | 0.635 | 0.676 | 0.725 | 0.773 | 0.824 |
| d2 | 0.728 | 0.745 | 0.762 | 0.785 | 0.815 |
| Hao | 0.626 | 0.594 | 0.604 | 0.573 | 0.571 |
| Manhattan | 0.692 | 0.741 | 0.766 | 0.791 | 0.823 |
| Chebyshev | 0.640 | 0.624 | 0.612 | 0.618 | 0.636 |
| Euclidean | 0.733 | 0.750 | 0.767 | 0.793 | 0.824 |
| Teeling | 0.625 | 0.600 | 0.607 | 0.574 | 0.577 |
| EuF | 0.640 | 0.648 | 0.720 | 0.774 | 0.845 |
| Contig Length 1,000 bp | | | | | |
| K | 6 | 7 | 8 | 9 | 10 |
| d2* | 0.728 | 0.764 | 0.798 | 0.832 | 0.862 |
| d2S | 0.670 | 0.726 | 0.781 | 0.825 | 0.866 |
| d2 | 0.750 | 0.766 | 0.786 | 0.809 | 0.837 |
| Hao | 0.682 | 0.641 | 0.654 | 0.630 | 0.616 |
| Manhattan | 0.680 | 0.748 | 0.785 | 0.814 | 0.848 |
| Chebyshev | 0.663 | 0.654 | 0.645 | 0.643 | 0.642 |
| Euclidean | 0.753 | 0.772 | 0.788 | 0.816 | 0.847 |
| Teeling | 0.684 | 0.645 | 0.653 | 0.632 | 0.620 |
| EuF | 0.684 | 0.668 | 0.755 | 0.816 | 0.875 |
| Contig Length 10,000 bp | | | | | |
| K | 6 | 7 | 8 | 9 | 10 |
| d2* | 0.866 | 0.873 | 0.901 | 0.928 | 0.939 |
| d2S | 0.827 | 0.857 | 0.895 | 0.922 | 0.937 |
| d2 | 0.780 | 0.803 | 0.826 | 0.846 | 0.868 |
| Hao | 0.868 | 0.806 | 0.864 | 0.858 | 0.834 |
| Manhattan | 0.713 | 0.729 | 0.760 | 0.840 | 0.884 |
| Chebyshev | 0.753 | 0.749 | 0.738 | 0.716 | 0.718 |
| Euclidean | 0.791 | 0.813 | 0.836 | 0.854 | 0.881 |
| Teeling | 0.861 | 0.814 | 0.863 | 0.863 | 0.845 |
| EuF | 0.846 | 0.814 | 0.873 | 0.895 | 0.906 |

Table S5. The accession number of the 18 temperate phages and 108 lytic phages used for novel phages.

| Accession Number | Lifestyle | Accession Number | Lifestyle |
| --- | --- | --- | --- |
| NC_013197 | Lytic | NC_004462 | Temperate |
| NC_023502 | Lytic | NC_023559 | Temperate |
| NC_023557 | Lytic | NC_023560 | Temperate |
| NC_023566 | Lytic | NC_023571 | Temperate |
| NC_023569 | Lytic | NC_023591 | Temperate |
| NC_023590 | Lytic | NC_023734 | Temperate |
| NC_023592 | Lytic | NC_024369 | Temperate |
| NC_023594 | Lytic | NC_025422 | Temperate |
| NC_023610 | Lytic | NC_025428 | Temperate |
| NC_023614 | Lytic | NC_025431 | Temperate |
| NC_023718 | Lytic | NC_027334 | Temperate |
| NC_023859 | Lytic | NC_028743 | Temperate |
| NC_023863 | Lytic | NC_028768 | Temperate |
| NC_024149 | Lytic | NC_028834 | Temperate |
| NC_024329 | Lytic | NC_028954 | Temperate |
| NC_024358 | Lytic | NC_029046 | Temperate |
| NC_024711 | Lytic | NC_029078 | Temperate |
| NC_025429 | Lytic | NC_029119 | Temperate |
| NC_025436 | Lytic |  |  |
| NC_025439 | Lytic |  |  |
| NC_025458 | Lytic |  |  |
| NC_025459 | Lytic |  |  |
| NC_025462 | Lytic |  |  |
| NC_025463 | Lytic |  |  |
| NC_025466 | Lytic |  |  |
| NC_025467 | Lytic |  |  |
| NC_025470 | Lytic |  |  |
| NC_025471 | Lytic |  |  |
| NC_025822 | Lytic |  |  |
| NC_026013 | Lytic |  |  |
| NC_026582 | Lytic |  |  |
| NC_026610 | Lytic |  |  |
| NC_026612 | Lytic |  |  |
| NC_026613 | Lytic |  |  |
| NC_026665 | Lytic |  |  |
| NC_027120 | Lytic |  |  |
| NC_027125 | Lytic |  |  |
| NC_027294 | Lytic |  |  |
| NC_027295 | Lytic |  |  |
| NC_027296 | Lytic |  |  |
| NC_027333 | Lytic |  |  |
| NC_027336 | Lytic |  |  |
| NC_027342 | Lytic |  |  |
| NC_027346 | Lytic |  |  |
| NC_027348 | Lytic |  |  |
| NC_027354 | Lytic |  |  |
| NC_027357 | Lytic |  |  |
| NC_027358 | Lytic |  |  |
| NC_027359 | Lytic |  |  |
| NC_027361 | Lytic |  |  |
| NC_027362 | Lytic |  |  |
| NC_027363 | Lytic |  |  |
| NC_027367 | Lytic |  |  |
| NC_027370 | Lytic |  |  |
| NC_027371 | Lytic |  |  |
| NC_027373 | Lytic |  |  |
| NC_027377 | Lytic |  |  |
| NC_027385 | Lytic |  |  |
| NC_027386 | Lytic |  |  |
| NC_027389 | Lytic |  |  |
| NC_027391 | Lytic |  |  |
| NC_027400 | Lytic |  |  |
| NC_027401 | Lytic |  |  |
| NC_027633 | Lytic |  |  |
| NC_027634 | Lytic |  |  |
| NC_027635 | Lytic |  |  |
| NC_027636 | Lytic |  |  |
| NC_027637 | Lytic |  |  |
| NC_027638 | Lytic |  |  |
| NC_027639 | Lytic |  |  |
| NC_027640 | Lytic |  |  |
| NC_027641 | Lytic |  |  |
| NC_027642 | Lytic |  |  |
| NC_027643 | Lytic |  |  |
| NC_027644 | Lytic |  |  |
| NC_027645 | Lytic |  |  |
| NC_027646 | Lytic |  |  |
| NC_027647 | Lytic |  |  |
| NC_027648 | Lytic |  |  |
| NC_027987 | Lytic |  |  |
| NC_028651 | Lytic |  |  |
| NC_028676 | Lytic |  |  |
| NC_028693 | Lytic |  |  |
| NC_028694 | Lytic |  |  |
| NC_028776 | Lytic |  |  |
| NC_028805 | Lytic |  |  |
| NC_028831 | Lytic |  |  |
| NC_028865 | Lytic |  |  |
| NC_028899 | Lytic |  |  |
| NC_028950 | Lytic |  |  |
| NC_028966 | Lytic |  |  |
| NC_028967 | Lytic |  |  |
| NC_028993 | Lytic |  |  |
| NC_028994 | Lytic |  |  |
| NC_029005 | Lytic |  |  |
| NC_029006 | Lytic |  |  |
| NC_029012 | Lytic |  |  |
| NC_029014 | Lytic |  |  |
| NC_029021 | Lytic |  |  |
| NC_029028 | Lytic |  |  |
| NC_029047 | Lytic |  |  |
| NC_029061 | Lytic |  |  |
| NC_029065 | Lytic |  |  |
| NC_029068 | Lytic |  |  |
| NC_029075 | Lytic |  |  |
| NC_029106 | Lytic |  |  |
| NC_029118 | Lytic |  |  |
| NC_030476 | Lytic |  |  |

Table S6. The True Positive Rates (TPR) for classifying the lifestyles for contigs of 3,000bp from the 325 phage genomes identified after 1 January 2017 using different dissimilarity measures. The TPRs for temperate and lytic phage contigs were both larger than 60% are shown in this Table. TPR1 is the Ture Positive Rate for temperate phage contigs. TPR2 is the True Positive Rate for lytic phage contigs. TPR is the Ture Positive Rate for all the phage contigs.

|  | K | Markov Order | TPR1 | TPR1 | TPR |
| --- | --- | --- | --- | --- | --- |
| d2s | 7 | 1 | 0.680 | 0.720 | 0.713 |
| d2s | 8 | 2 | 0.732 | 0.625 | 0.642 |
| d2s | 8 | 3 | 0.639 | 0.694 | 0.685 |
| d2s | 9 | 1 | 0.835 | 0.697 | 0.718 |
| d2s | 9 | 2 | 0.770 | 0.626 | 0.649 |
| d2s | 9 | 3 | 0.612 | 0.750 | 0.728 |
| d2s | 10 | 1 | 0.774 | 0.776 | 0.776 |
| d2s | 10 | 2 | 0.673 | 0.781 | 0.764 |
| d2s | 10 | 3 | 0.755 | 0.793 | 0.787 |
| d2 | 7 |  | 0.966 | 0.639 | 0.690 |
| d2 | 8 |  | 0.961 | 0.653 | 0.701 |
| d2 | 9 |  | 0.954 | 0.670 | 0.714 |
| d2 | 10 |  | 0.943 | 0.700 | 0.738 |
| Manhattan | 6 |  | 0.962 | 0.686 | 0.729 |
| Chebyshev | 10 |  | 0.654 | 0.627 | 0.631 |
| Euclidean | 7 |  | 0.956 | 0.646 | 0.695 |
| Euclidean | 8 |  | 0.939 | 0.663 | 0.706 |
| Euclidean | 9 |  | 0.917 | 0.686 | 0.722 |
| Euclidean | 10 |  | 0.873 | 0.724 | 0.748 |

Table S7. The True Positive Rates (TPR) for classifying the lifestyles for contigs of 1,000bp from the 325 phage genomes identified after 1 January 2017 using different dissimilarity measures. The TPRs for temperate and lytic phage contigs were both larger than 60% are shown in this Table. TPR1 is the Ture Positive Rate for temperate phage contigs. TPR2 is the True Positive Rate for lytic phage contigs. TPR is the Ture Positive Rate for all the phage contigs.

|  | K | Markov Order | TPR1 | TPR1 | TPR |
| --- | --- | --- | --- | --- | --- |
| d2s | 6 | 2 | 0.710 | 0.613 | 0.628 |
| d2s | 7 | 2 | 0.784 | 0.636 | 0.659 |
| d2s | 8 | 2 | 0.820 | 0.687 | 0.708 |
| d2s | 8 | 3 | 0.692 | 0.757 | 0.747 |
| d2s | 9 | 1 | 0.914 | 0.737 | 0.764 |
| d2s | 9 | 2 | 0.864 | 0.670 | 0.700 |
| d2s | 9 | 3 | 0.649 | 0.809 | 0.784 |
| d2s | 10 | 1 | 0.847 | 0.799 | 0.806 |
| d2s | 10 | 2 | 0.743 | 0.815 | 0.804 |
| d2s | 10 | 3 | 0.819 | 0.812 | 0.813 |
| d2 | 6 |  | 0.978 | 0.652 | 0.703 |
| d2 | 7 |  | 0.975 | 0.665 | 0.713 |
| d2 | 8 |  | 0.971 | 0.684 | 0.728 |
| d2 | 9 |  | 0.965 | 0.712 | 0.751 |
| d2 | 10 |  | 0.954 | 0.754 | 0.785 |
| CVTree | 7 |  | 0.722 | 0.674 | 0.682 |
| Teeling | 8 |  | 0.613 | 0.640 | 0.636 |
| Manhattan | 6 |  | 0.975 | 0.656 | 0.706 |
| Manhattan | 7 |  | 0.982 | 0.675 | 0.723 |
| Chebyshev | 10 |  | 0.683 | 0.630 | 0.638 |
| Euclidean | 7 |  | 0.968 | 0.655 | 0.704 |
| Euclidean | 8 |  | 0.955 | 0.675 | 0.718 |
| Euclidean | 9 |  | 0.941 | 0.697 | 0.735 |
| Euclidean | 10 |  | 0.919 | 0.738 | 0.766 |
